# Visible-Light-Mediated Catalyst-Free [2+2] Cycloaddition Reaction for Dihydrocyclobuta[*b*]naphthalene-3,8-diones Synthesis under Mild Conditions

**DOI:** 10.3390/molecules28227654

**Published:** 2023-11-18

**Authors:** Hong-Bo Tan, Jia-Ying Zhou, Ying-Shan Liu, Tong Lei, Shi-Yu Wang, Shuang-Shuang Hu, Xu Zhang, Zhi-Gang Xu, Dian-Yong Tang, Zhong-Zhu Chen, Bo-Chu Wang

**Affiliations:** 1 National and Local Joint Engineering Research Center of Targeted and Innovative Therapeutics, Chongqing Engineering Laboratory of Targeted and Innovative Therapeutics, Chongqing Key Laboratory of Kinase Modulators as Innovative Medicine, Chongqing Collaborative Innovation Center of Targeted and Innovative Therapeutics, College of Pharmacy & IATTI, Chongqing University of Arts and Sciences, Chongqing 402160, China; 18723843062@139.com (J.-Y.Z.); 18883138277@163.com (Z.-Z.C.); 2 Key Laboratory of Biorheological Science and Technology, Ministry of Education, College of Bioengineering, Chongqing University, Chongqing 400030, China; 3 Chongqing Academy of Chinese Materia Medica, Chongqing 400065, China

**Keywords:** green chemistry, photocycloaddition, visible-light photocatalysis, [2+2] cycloaddition, dihydrocyclobuta[*b*]naphthalene-3,8-diones

## Abstract

A facile and efficient visible-light-mediated method for directly converting 1,4-naphthoquinones into dihydrocyclo-buta[*b*]naphthalene-3,8-diones (DHCBNDOs) under mild and clean conditions without using any photocatalysts is reported. This approach exhibited favorable compatibility with functional groups and afforded a series of DHCBNDOs with excellent regioselectivity and high yields. Moreover, detailed mechanism studies were carried out both experimentally and theoretically. The readily accessible, low-cost and ecofriendly nature of the developed strategy will endow it with attractive applications in organic and medicinal chemistry.

## 1. Introduction

**Figure 1:**
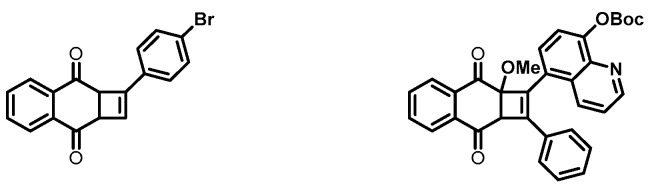
Representative examples with the skeleton of DHCBNDO.

The development of green, concise and mild synthetic methodologies for extensively used organic compounds is one of the most essential tasks in the area of organic synthesis. Fused cyclobutene is a type of unique and intriguing structural motif in organic chemistry, which has attracted considerable attention in recent years because it is luxuriant in nature and shows multiple biological activities [1,2,3,4,5,6,7,8,9]. Furthermore, owing to the high chemical reactivity from the inherent and high ring strain, the fused cyclobutenes experience diverse transformations, ring-opening and ring-expansion reactions in particular, providing distinctive approaches to the construction of complex organic molecules [10,11,12,13]. Dihydrocyclobuta [*b*] naphthalene-3,8-diones (DHCBNDOs) containing fused cyclobutene structures are highly valued scaffolds that occur in many bioactive compounds (Figure 1) and, in particular, operate as lead compounds for SARS-CoV-19 management, [14] miR-1 inhibitors [15], and microRNAs activators [16]. A great deal of attention has been devoted to developing better strategies for synthesizing DHCBNDOs and their analogs, of which [2+2] cycloaddition is generally considered to be one of the most forthright and efficient methods [17,18,19,20,21]. The DHCBNDOs, an interesting scaffold for developing new drugs for SARS-CoV-19, coronary artery diseases, heart attacks and miRNA-based tumors, are under-represented in medicinal chemistry [14,15,16], and this potentially results from deficiencies in extant synthetic methodologies.

**Scheme 1:**
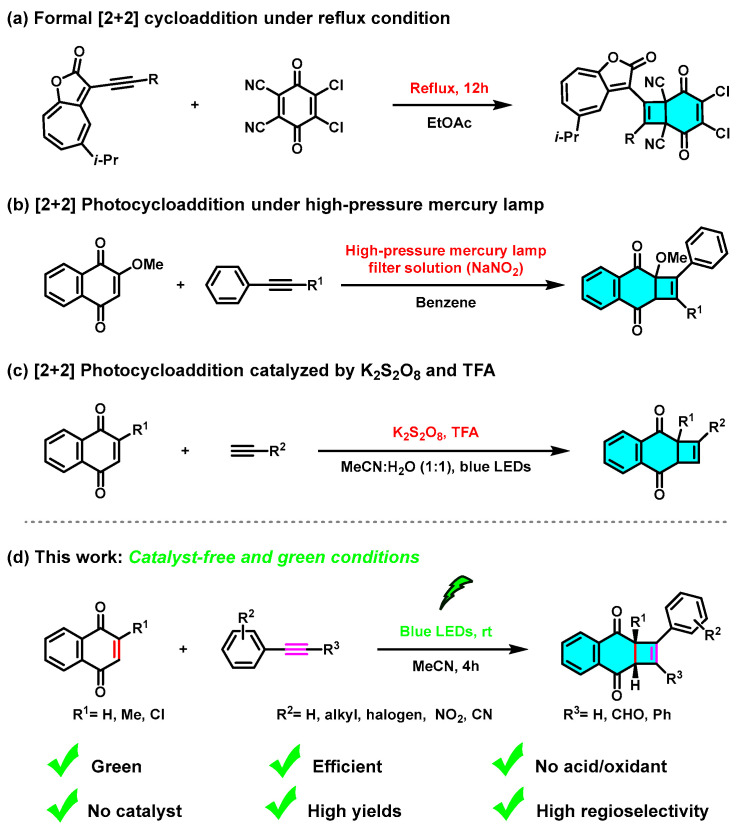
[2+2] cycloaddition reaction for the synthesis of DHCBNDOs.

As shown in Scheme 1, the DHCBNDO system has only recently been synthesized by formal [2+2] cycloaddition under reflux condition, as reported by Diederich, Morita and coworkers [22,23,24]. Some great improvements via [2+2] photocycloaddition have been made by Zhang’s (high-pressure mercury lamp) [15] and Shah’s (visible-light-mediated [2+2] cycloaddition) groups [25]. However, the reported methods severely relied on some unfriendly conditions, such as heating, high-pressure mercury lamp, acid catalyst and oxidants, which created some drawbacks in these strategies in terms of environmental protection, safety, economy, reaction selectivity and functional group tolerance. Although significant progress has been achieved in the synthesis of DHCBNDO systems over previous decades, the development of approaches for preparing DHCBNDOs in an efficient, green and facile way is an ongoing challenge that urgently needs to be addressed.

To supplement our initial research in the synthesis of tricyclic or polycyclic fused organic compounds using photocatalytic reactions [26,27,28,29], we would like to report a green, facile and visible-light-mediated [2+2] cycloaddition reaction for DHCBNDOs synthesis at ambient temperature. We believe this approach not only represents a mild, clean and economical method for the preparation of DHCBNDOs, but also exhibits promising prospects in synthetic chemistry, such as constructing organic molecules of complex structures.

## 2. Results and Discussion

In our pilot experiment, menadione **1a** (1 mmol) and phenylacetylene **2a** (1 mmol) were chosen as model substrates in solvet MeCN, which was irradiated under blue LEDs (460 nm) for 3 h. The target tricyclic framework compound 8a-methyl-1-phenyl-2a,8a-dihydrocyclobuta[*b*]naphthalene-3,8-dione **3aa** was obtained in 81% yield without any catalyst (Table 1, entry 1). Furthermore, the use of several different solvents, including DCM, acetone, dioxane, chlorobenzene, MeOH, THF, toluene, and DCE, did not give a better result than MeCN (Table 1, entries 220139, respectively). However, when a solvent of MeCN was used with irradiation for 4 h, the yield of **3aa** was improved to 86%. (Table 1, entry 10). Increasing the reaction time to 5h or increasing the equivalent of **2a** to 1.4eq failed to give any better results (Table 1, entries 11–14, respectively). And the control experiments of no light and different wavelength (365 nm) did not give better results (Table 1, entries 15–16, respectively). Therefore, the optimized reaction conditions for this [2+2] cycloaddition reaction turned out to be those obtained using MeCN as the solvent (0.1 mmol/L) under blue LEDs (460 nm) irradiation for 4h.

**Scheme 2:**
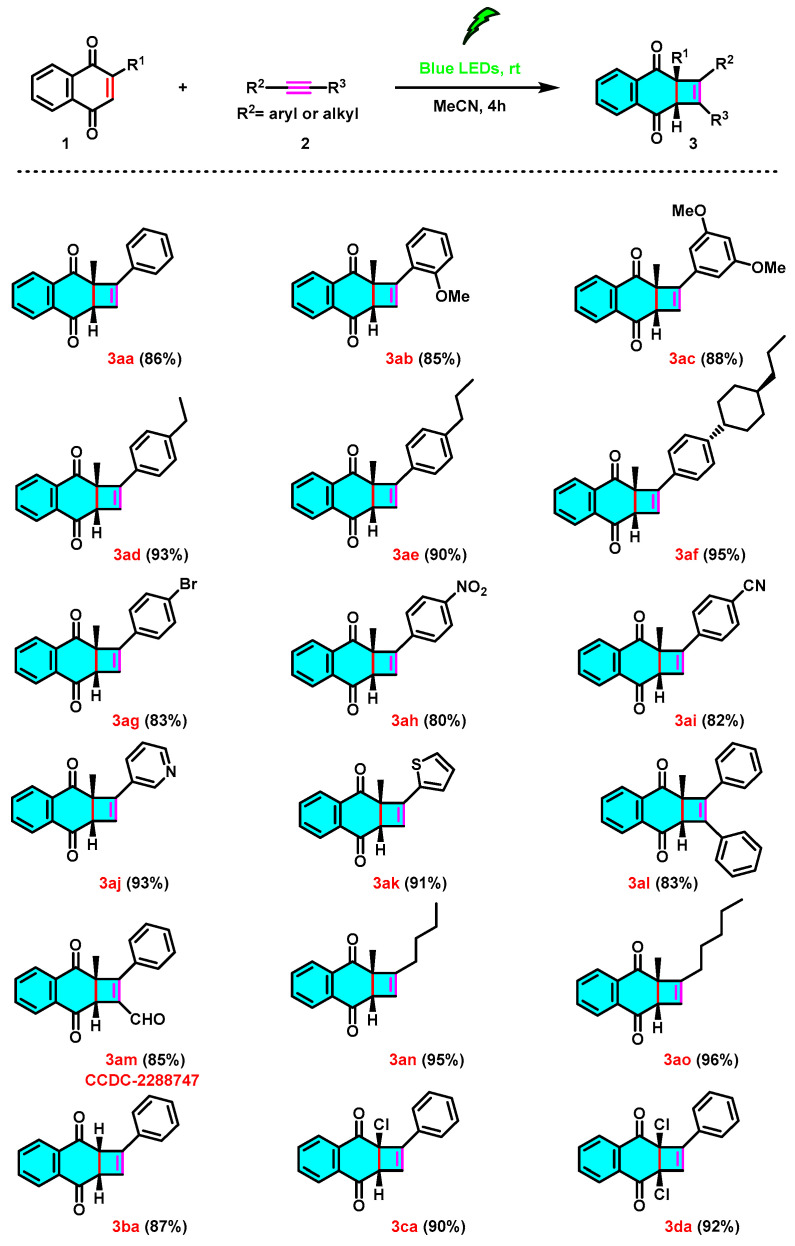
Exploration of substrate scope.

**Figure 2:**
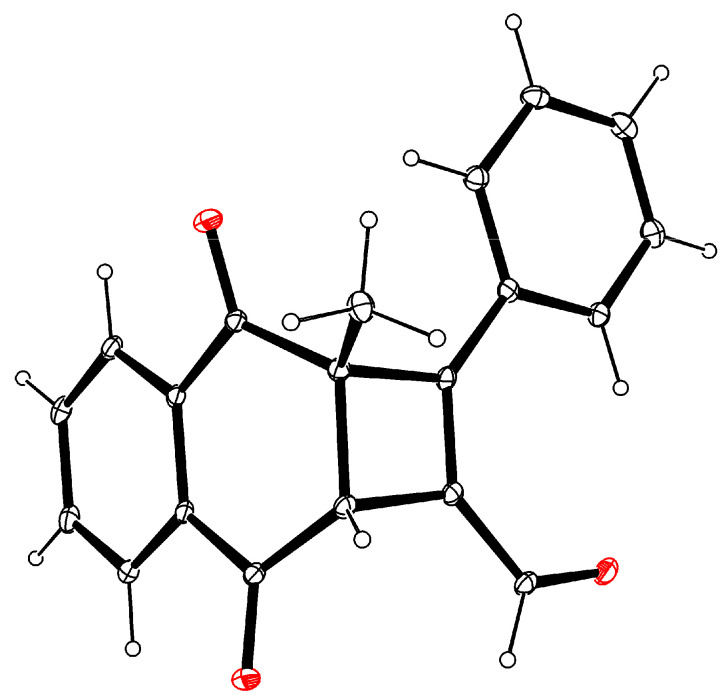
ORTEP diagram of crystal structures of **3am**. (CCDC-2288747).

With the optimized conditions in hand, a survey of the substrate scope was carried out by varying 1,4-naphthoquinone and alkyne compounds (Scheme 2). Phenylacetylene **2a** provided the corresponding product **3aa** in very good yields under optimized reaction conditions. And the variability of the phenylacetylene was checked using substituted alkyne **2**. The phenylacetylene **2** tethered with an electron-donating group, such as -OMe, 3,5-OMe, -Et, -Pr, and -cyclohexyl, also underwent [2+2] cycloaddition reactions with very good yields (**3ab**–**3af**). Similarly, **2** with electron-withdrawing groups, such as -Br, -NO_2_, and -CN, also underwent [2+2] cycloaddition reactions with very good yields (**3ag**–**3ai**). Alkyne derivatives with heterocycle substituents were also found to be suitable for this [2+2] cycloaddition reaction (**3aj** and **3ak**), and non-terminal alkynes with -CHO and -Ph groups were also suitable substrates, giving **3al** and **3am** in very good yields. Similarly, aliphatic alkynes also gave the corresponding [2+2] cycloaddition products (**3an** and **3ao**) in excellent yield, and 1,4-naphthoquinones bearing electron-withdrawing substituents (-H, -Cl, and -2,3Cl) did the same for corresponding products (**3ba**–**3da**) in high yields (87–92%). These different groups did not significantly affect the yields, probably due to the weak influence of electronic effects. It is noteworthy that the reaction proceeded with excellent regioselectivity due to the trans-product being hard to access because of the ring strain, giving target *cis*-8a-methyl-1-phenyl-2a,8a-dihydrocyclobuta[*b*]naphthalene-3,8-diones **3**. The proton NMR coupling signals (two doublets) from the two cyclobutene protons proved that -Me and -Ph groups were adjacent to each other (Supplementary Figure S1). The single-crystal X-ray diffraction (**CCDC-2288747**) further confirmed the *cis*-structure (Figure 2). As a consequence, the NMR and X-ray analysis confirmed excellent regioselectivity of the visible-light-mediated [2+2] cycloaddition reaction.

**Scheme 3:**
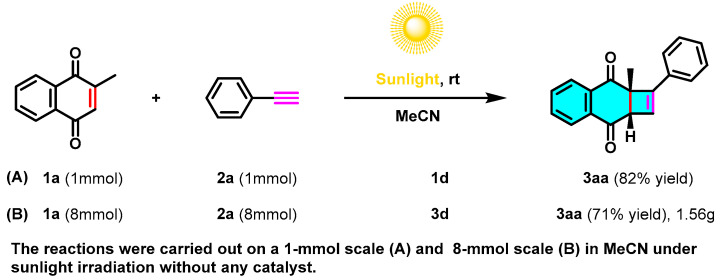
Sunlight irradiation and large-scale synthesis. (**A**) The 1-mmol-scale reaction under sunlight irradiation; (**B**) The 8-mmol-scale reaction under sunlight irradiation.

To evaluate the synthetic utility of our visible light-mediated approach, the reaction of **1a** and **2a** was carried out under sunlight irradiation (about 8 h per day), and the product **3aa** was given in 82% yield (Scheme 3A). In addition, a large-scale (8-mmol scale) preparation of **3aa** under sunlight irradiation was successful, with only a slightly decreased yield (71%), which confirmed the practicability of this strategy (Scheme 3B).

**Scheme 4:**
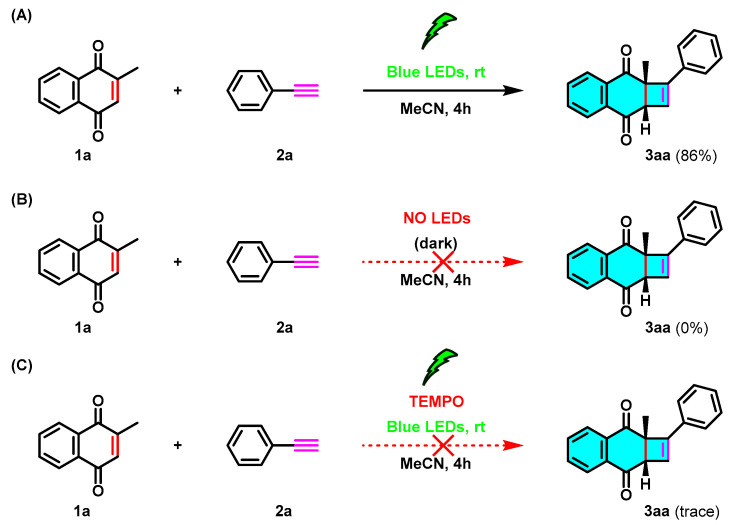
Control experiments. (**A**) The reaction under standard conditions; (**B**) The reaction under standard conditions without LEDs; (**C**) The reaction under standard conditions with addition of TEMPO.

**Scheme 5:**
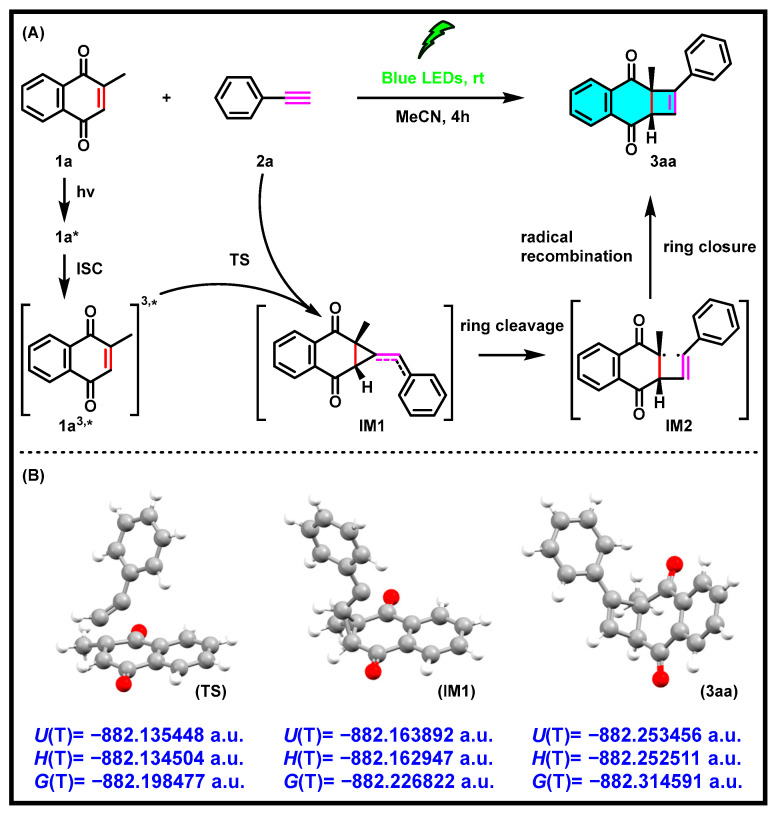
(**A**) Plausible pathway for the visible-light-mediated [2+2] cycloaddition reaction; (**B**) Optimized structures of stationary points along the pathway and corresponding calculated enthalpies and free energies (the numbers at the bottom of the Scheme 5B) at the um062x, 6-31g(d,p) theoretical level [30]. Symbol * means the lowest excited state.

After exploring the substrate scope and utility of this reaction, we focused on the study mechanism. In order to obtain full insight into this intermolecular [2+2]-cycloaddition reaction, some control experiments (Scheme 4) and theoretical calculations (Scheme 5) were conducted by using **1a** and **2a** as the model reactants. No reaction occurred in the dark environments (without blue LEDs) (Scheme 4B), which highlighted that the [2+2] cycloaddition reaction need to be triggered by blue LEDs. In addition, 2,2,6,6-tetramethyl-1-piperidinyloxy (with TEMPO as a radical scavenger) was added in the reaction of Scheme 4C, and only a trace amount of compound **3aa** was detected.

Based on the control experimental results, DFT-calculated results (see the computational details section) and previous studies of the [2+2] cycloaddition reaction [31], a possible mechanism for this visible-light-photocatalyzed [2+2] cycloaddition reaction was proposed, as shown in Scheme 5. First, the **1a**^3,*s^ was populated by intersystem crossing (ISC) of the singlet excited state 1a^*^ that resulted from visible light irradiation of ground state **1a**. The excited state of **1a**^3,*^ reacted with **2a** to deliver the cyclopropane intermediate (IM1) through a transition-state structure **TS** (IRC scan results in ESI, Supplementary Figure S40), and its free energy barrier was calculated as 13.4 kcal mol^−1^. The next step in the mechanistic pathway is the reaction of the ring cleavage of the cyclopropane with the possible formation of 1,4-biradical intermediate **IM2**. The intermediate **IM2** subsequently underwent intramolecular radical recombination, giving target ring-closure product **3aa**.

## 3. Materials and Methods

### 3.1. General Information

#### 3.1.1. Experimental Section

All chemical agents were purchased from chemical manufacturers (Bide and Energy Chemical) and directly used without any further purification. Thin layer chromatography was carried out on glass silica gel GF254 plates. Melting points were detected by a digital melting point apparatus (XT-5A). The NMR tests (1D-NMR and 2D-NMR) were conducted on a Bruker spectrometer (Avance 400). HRMS data were obtained from a Thermo Fisher mass spectrometer (Q-Exactive) in ESI mode.

#### 3.1.2. Computational Section

Geometrical optimization was carried out at the um062x 6-31g(d,p) theoretical level using the SCRF model (MeCN as solvent) [32,33,34]. Frequency analysis and the thermodynamic correctional data were obtained at the same level. In addition, the IRC pathways [35] calculations were also performed for transition state (TS) to identify whether the transition state can connect the reactants and the key intermediate (IM1). All of the computational experiments were conducted by using the Gaussian 16 program package [36].

### 3.2. General Procedure for the Preparation of DHCBNDOs

In a 15 mL tube, 1.0 mmol 1,4-naphthoquinone derivatives **1** and 1.0 mmol alkyne derivatives **2** were dissolved in 10 mL of MeCN. The solution was irradiated by blue LEDs (460 nm) at room temperature for 4 h. After completion, the reaction solution was evaporated in a vacuum which was purified by a Biotage Flash (medium-pressure chromatography) using a mixed solvent of hexane (HEX) and ethyl acetate (EA) (5–30% EA). The products **3** were characterized by proton NMR, carbon NMR and HRMS spectroscopy.

*(2aS,8aS)-8a-methyl-1-phenyl-2a,8a-dihydrocyclobuta[b]naphthalene-3,8-dione* **(3aa):** Yellow solid, yield 86%, m.p. 154–156 °C; ^1^H NMR (400 MHz, CDCl_3_): δ (ppm) 1.83 (s, 3H, CH_3_), 3.78 (d, 1H, J = 1.2 Hz, CH), 6.55 (d, 1H, J = 2.0 Hz, =CH), 7.27–7.34 (m, 3H, Ar-H), 7.49–7.51 (m, 2H, Ar-H), 7.68–7.71 (m, 2H, Ar-H), 8.01–8.06 (m, 2H, Ar-H); ^13^C NMR (100 MHz, CDCl_3_): δ (ppm) 19.8, 57.2, 57.5, 125.5, 127.0, 127.6, 128.0, 128.6, 129.1, 131.6, 133.7, 133.7, 134.4, 134.5, 153.4, 196.6, 198.4; HRMS (ESI), m/z calcd 275.1067 for C_19_H_15_O_2_ [M+H]^+^, found 275.1069.

*(2aS,8aS)-1-(2-methoxyphenyl)-8a-methyl-2a,8a-dihydrocyclobuta[b]naphthalene-3,8-dione* **(3ab)**: Faint yellow solid, yield 85%, 152–154 °C; ^1^H NMR (400 MHz, CDCl_3_): δ (ppm) 1.74 (s, 3H, CH_3_), 3.69 (s, 3H, OCH_3_), 3.72 (d, 1H, J = 1.6 Hz, CH), 6.54 (d, 1H, J = 1.6 Hz, =CH), 6.72 (d, 1H, J = 8.0 Hz, Ar-H), 6.85–6.88 (m, 1H, Ar-H), 7.12–7.16 (m, 1H, Ar-H), 7.48–7.59 (m, 3H, Ar-H); ^13^C NMR (100 MHz, CDCl_3_): δ (ppm) 20.0, 55.0, 57.5, 59.0, 110.4, 120.5, 120.6, 126.9, 127.8, 127.9, 127.9, 129.8, 132.9, 133.7, 134.0, 134.2, 134.3, 149.9, 158.9, 196.9, 199.0; HRMS (ESI), m/z calcd 305.1172 for C_20_H_17_O_3_ [M+H]^+^, found 305.1176.

*(2aS,8aS)-1-(3,5-dimethoxyphenyl)-8a-methyl-2a,8a-dihydrocyclobuta[b]naphthalene-3,8-dione* **(3ac):** Faint yellow solid, yield 88%, 151–153 °C; ^1^H NMR (400 MHz, CDCl_3_): δ (ppm) 1.74 (s, 3H, CH_3_), 3.68 (d, 1H, J = 1.6 Hz, CH), 3.70 (s, 6H, OCH_3_), 6.31 (t, 1H, J = 2.0 Hz, Ar-H), 6.46 (d, 1H, J = 1.6 Hz, =CH), 6.58 (t, 2H, J = 2.0 Hz, Ar-H), 7.61–7.64 (m, 2H, Ar-H), 7.93–7.99 (m, 2H, Ar-H); ^13^C NMR (100 MHz, CDCl_3_): δ (ppm) 19.9, 55.4, 57.1, 57.4, 101.6, 103.4, 127.0, 127.6, 128.0, 128.1, 133.2, 133.6, 133.7, 134.4, 134.5, 153.3, 161.0, 196.6, 198.3; HRMS (ESI), m/z calcd 335.1278 for C_21_H_19_O_4_ [M+H]^+^, found 335.1280.

*(2aS,8aS)-1-(4-ethylphenyl)-8a-methyl-2a,8a-dihydrocyclobuta[b]naphthalene-3,8-dione* **(3ad)**: Yellow solid, yield 93%, 155–157 °C; ^1^H NMR (400 MHz, CDCl_3_): δ (ppm) 1.20 (t, 3H, J = 7.6 Hz, CH_3_), 1.83 (s, 3H, CH_3_), 2.61 (q, 2H, J = 7.6 Hz, CH_2_), 3.77 (d, 1H, J = 1.6 Hz, CH), 6.49 (d, 1H, J = 1.6 Hz, =CH), 7.16 (d, 2H, J = 8.4 Hz, Ar-H), 7.42 (d, 2H, J = 8.4 Hz, Ar-H), 7.69–7.72 (m, 2H, Ar-H), 8.02–8.06 (m, 2H, Ar-H); ^13^C NMR (100 MHz, CDCl_3_): δ (ppm) 15.4, 19.8, 28.8, 57.1, 57.5, 125.6, 126.4, 127.0, 128.0, 128.1, 129.2, 133.7, 133.8, 134.3, 134.4, 145.6, 153.5, 196.9, 198.6; HRMS (ESI), m/z calcd 303.1380 for C_21_H_19_O_2_ [M+H]^+^, found 303.1385.

*(2aS,8aS)-8a-methyl-1-(4-propylphenyl)-2a,8a-dihydrocyclobuta[b]naphthalene-3,8-dione* **(3ae)**: Yellow solid, yield 90%, 154–156 °C; ^1^H NMR (400 MHz, CDCl_3_): δ (ppm) 0.84 (t, 3H, J = 7.2 Hz, CH_3_), 1.53 (q, 2H, J = 7.6 Hz, CH_2_), 1.75 (s, 3H, CH_3_), 2.47 (t, 2H, CH_2_), 3.70 (d, 1H, J = 1.6 Hz, CH), 6.40 (d, 1H, J = 1.6 Hz, =CH), 7.06 (d, 2H, J = 8.0 Hz, Ar-H), 7.34 (d, 2H, J = 8.4 Hz, Ar-H), 7.62–7.65 (m, 2H, Ar-H), 7.94–7.99 (m, 2H, Ar-H); ^13^C NMR (100 MHz, CDCl_3_): δ (ppm) 13.8, 19.8, 24.4, 37.9, 57.1, 57.5, 125.5, 126.4, 127.0, 128.0, 128.7, 129.2, 133.7, 133.8, 134.3, 134.4, 144.0, 153.5, 196.9, 198.6; HRMS (ESI), m/z calcd 317.1536 for C_22_H_21_O_2_ [M+H]^+^, found 317.1539.

*(2aS,8aS)-8a-methyl-1-(4-((1s,4S)-4-propylcyclohexyl)phenyl)-2a,8a-dihydrocyclobuta[b]naphthalene-3,8-dione* **(3af):** Yellow solid, yield 95%, 149–151 °C; ^1^H NMR (400 MHz, CDCl_3_): δ (ppm) 0.89 (t, 3H, J = 7.2 Hz, CH_3_), 1.04–1.07 (m, 2H, CH_2_), 1.18–1.22 (m, 2H, CH_2_), 1.28–1.42 (m, 6H, CH_2_), 1.82 (s, 3H, CH_3_), 1.85 (s, 3H, CH_3_), 2.40–2.46 (m, 1H, CH), 3.76 (d, 1H, J = 1.6 Hz, CH), 6.48 (d, 1H, J = 1.6 Hz, =CH), 7.17 (d, 2H, J = 8.4 Hz, Ar-H), 7.42 (d, 2H, J = 8.4 Hz, Ar-H), 7.68–7.72 (m, 2H, Ar-H), 8.01–8.06 (m, 2H, Ar-H); ^13^C NMR (100 MHz, CDCl_3_): δ (ppm) 14.4, 19.9, 20.0, 33.5, 34.1, 37.0, 39.7, 44.6, 57.1, 57.5, 125.5, 126.4, 127.0, 128.0, 129.3, 133.7, 133.7, 134.3, 134.4, 149.2, 153.5, 196.8, 198.5; HRMS (ESI), m/z calcd 399.2319 for C_28_H_31_O_2_ [M+H]^+^, found 399.2323.

*(2aS,8aS)-1-(4-bromophenyl)-8a-methyl-2a,8a-dihydrocyclobuta[b]naphthalene-3,8-dione* **(3ag)**: Yellow solid, yield 83%, 165–167 °C; ^1^H NMR (400 MHz, CDCl_3_): δ (ppm) 1.72 (s, 3H, CH_3_), 3.68 (d, 1H, J = 1.6 Hz, CH), 6.48 (d, 1H, J = 2.0 Hz, =CH), 7.28 (d, 2H, J=8.8 Hz, Ar-H), 7.36 (d, 2H, J = 8.8 Hz, Ar-H), 7.62–7.65 (m, 2H, Ar-H), 7.94–7.98 (m, 2H, Ar-H); ^13^C NMR (100 MHz, CDCl_3_): δ (ppm) 19.8, 57.1, 57.5, 123.3, 127.1, 127.1, 128.0, 128.3, 130.3, 131.9, 133.5, 133.6, 134.5, 134.6, 152.2, 196.2, 198.2; HRMS (ESI), m/z calcd 353.0172 for C_19_H_14_BrO_2_ [M+H]^+^, found 353.0176.

*(2aS,8aS)-8a-methyl-1-(4-nitrophenyl)-2a,8a-dihydrocyclobuta[b]naphthalene-3,8-dione* **(3ah)**: Yellow solid, yield 80%, 173–175 °C; ^1^H NMR (400 MHz, CDCl_3_): δ (ppm) 1.78 (s, 3H, CH_3_), 3.78 (d, 1H, J = 1.6 Hz, CH), 6.72 (d, 1H, J = 2.0 Hz, =CH), 7.62 (d, 2H, J = 8.8 Hz, Ar-H), 7.68–7.71 (m, 2H, Ar-H), 7.99–8.03 (m, 2H, Ar-H), 8.11 (d, 2H, J = 8.8 Hz, Ar-H); ^13^C NMR (100 MHz, CDCl_3_): δ (ppm) 19.9, 57.2, 57.7, 124.0, 126.4, 127.2, 128.2, 133.4, 133.6, 134.8, 134.8, 137.1, 147.6, 151.1, 195.4, 197.6; HRMS (ESI), m/z calcd 320.0917 for C_19_H_14_NO_4_ [M+H]^+^, found 320.0921.

*4-((2aS,8aS)-8a-methyl-3,8-dioxo-2a,3,8,8a-tetrahydrocyclobuta[b]naphthalen-1-yl)benzonitrile* **(3ai)**: Yellow solid, yield 82%, 170–172 °C; ^1^H NMR (400 MHz, CDCl_3_): δ (ppm) 1.75 (s, 3H, CH_3_), 3.75 (br s, 1H, CH), 6.66 (br s, 1H, =CH), 7.54 (br s, 4H, Ar-H), 7.66–7.68 (m, 2H, Ar-H), 7.96–8.01 (m, 2H, Ar-H); ^13^C NMR (100 MHz, CDCl_3_): δ (ppm) 19.9, 57.2, 57.6, 112.3, 118.5, 126.1, 127.2, 128.1, 131.7, 132.4, 133.4, 133.6, 134.7, 134.7, 135.3, 151.4, 195.5, 197.7; HRMS (ESI), m/z calcd 300.1019 for C_20_H_14_NO_2_ [M+H]^+^, found 300.1022.

*(2aS,8aS)-8a-methyl-1-(pyridin-3-yl)-2a,8a-dihydrocyclobuta[b]naphthalene-3,8-dione* **(3aj)**: Yellow solid, yield 93%, 168–170 °C; ^1^H NMR (400 MHz, CDCl_3_): δ (ppm) 1.74 (s, 3H, CH_3_), 3.74 (d, 1H, J = 1.6 Hz, CH), 6.59 (d, 1H, J = 2.0 Hz, =CH), 6.17 (dd, 1H, J = 8.0 Hz, 4.8 Hz, Ar-H), 7.63–7.66 (m, 2H, Ar-H), 7.74 (dt, 1H, J = 8.0 Hz, 2.0 Hz, Ar-H), 7.94–7.99 (m, 2H, Ar-H), 8.39 (dd, 1H, J = 4.8 Hz, 1.6 Hz, Ar-H), 8.65 (d, 1H, J = 2.0 Hz, Ar-H); ^13^C NMR (100 MHz, CDCl_3_): δ (ppm) 19.8, 57.2, 57.7, 123.5, 127.1, 127.4, 128.0, 129.9, 132.7, 133.6, 134.6, 134.6, 147.0, 149.6, 150.5, 195.9, 197.8; HRMS (ESI), m/z calcd 276.1019 for C_18_H_14_NO_2_ [M+H]^+^, found 276.1024.

*(2aS,8aS)-8a-methyl-1-(thiophen-2-yl)-2a,8a-dihydrocyclobuta[b]naphthalene-3,8-dione* **(3ak)**: Faint yellow solid, yield 91%, 164–166 °C; ^1^H NMR (400 MHz, CDCl_3_): δ (ppm) 1.72 (s, 3H, CH_3_), 3.71 (d, 1H, J = 1.6 Hz, CH), 6.17 (d, 1H, J = 1.6 Hz, =CH), 6.90 (dd, 1H, J = 4.8 Hz, 3.6 Hz, Ar-H), 7.18–7.20 (m, 2H, Ar-H), 7.61–7.64 (m, 2H, Ar-H), 7.94–7.99 (m, 2H, Ar-H); ^13^C NMR (100 MHz, CDCl_3_): δ (ppm) 19.7, 57.5, 57.8, 125.0, 126.7, 126.8, 127.2, 127.7, 128.0, 133.4, 133.8, 134.4, 134.5, 134.5, 147.6, 196.2, 197.5; HRMS (ESI), m/z calcd 281.0631 for C_17_H_13_O_2_S [M+H]^+^, found 281.0635.

*(2aS,8aS)-2a-methyl-1,2-diphenyl-2a,8a-dihydrocyclobuta[b]naphthalene-3,8-dione* **(3al)**: Yellow solid, yield 83%, 160–162 °C; ^1^H NMR (400 MHz, CDCl_3_): δ (ppm) 1.80 (s, 3H, CH_3_), 4.21 (s, 1H, CH), 7.28–7.37 (m, 6H, Ar-H), 7.51–7.55 (m, 4H, Ar-H), 7.68–7.77 (m, 2H, Ar-H), 7.93 (dd, 1H, J = 7.6 Hz, 1.2 Hz, Ar-H), 8.13 (dd, 1H, J = 7.6 Hz, 1.2 Hz, Ar-H); ^13^C NMR (100 MHz, CDCl_3_): δ (ppm) 19.3, 55.5, 58.5, 126.9, 127.0, 127.0, 127.9, 128.5, 128.7, 128.9, 128.9, 132.8, 132.8, 133.9, 134.0, 134.3, 134.5, 140.6, 145.0, 196.7, 198.6; HRMS (ESI), m/z calcd 351.1380 for C_25_H_19_O_2_ [M+H]^+^, found 351.1385.

*(2aS,8aS)-2a-methyl-3,8-dioxo-2-phenyl-2a,3,8,8a-tetrahydrocyclobuta[b]naphthalene-1-carbaldehyde* (**3am**): Faint yellow solid, yield 85%, 173–175 °C; ^1^H NMR (400 MHz, CDCl_3_): δ (ppm) 1.81 (s, 3H, CH_3_), 4.00 (s, 1H, CH), 7.33–7.39 (m, 3H, Ar-H), 7.63–7.68 (m, 2H, Ar-H), 7.84 (dd, 2H, J = 8.0 Hz, 2.0 Hz, Ar-H), 7.94 (d, 1H, J = 7.6 Hz, Ar-H), 9.85 (s, 1H, CHO); ^13^C NMR (100 MHz, CDCl_3_): δ (ppm) 20.3, 56.2, 127.4, 128.0, 129.1, 129.3, 130.8, 132.0, 133.5, 133.8, 134.6, 134.9, 135.0, 161.1, 185.0, 194.4, 196.7; HRMS (ESI), m/z calcd 303.1016 for C_20_H_15_O_3_ [M+H]^+^, found 303.1020.

*(2aS,8aS)-1-butyl-8a-methyl-2a,8a-dihydrocyclobuta[b]naphthalene-3,8-dione* **(3an)**: Faint yellow oil, yield 95%; ^1^H NMR (400 MHz, CDCl_3_): δ (ppm) 0.83 (t, 3H, J = 7.2 Hz, CH_3_), 1.21–1.39 (m, 4H, CH_2_), 1.59 (s, 3H, CH_3_), 1.92–2.08 (m, 2H, CH_2_), 3.62 (d, 1H, J = 1.2 Hz, CH), 6.01 (d, 1H, J = 1.2 Hz, =CH), 7.74–7.76 (m, 2H, Ar-H), 8.03–8.10 (m, 2H, Ar-H); ^13^C NMR (100 MHz, CDCl_3_): δ (ppm) 13.7, 18.9, 22.3, 27.0, 27.5, 57.1, 58.0, 127.1, 127.6, 128.8, 133.4, 133.8, 134.3, 134.3, 159.1, 197.6, 198.1; HRMS (ESI), m/z calcd 255.1380 for C_17_H_19_O_2_ [M+H]^+^, found 255.1385.

*(2aS,8aS)-8a-methyl-1-pentyl-2a,8a-dihydrocyclobuta[b]naphthalene-3,8-dione* **(3ao)**: Faint yellow oil, yield 96%; ^1^H NMR (400 MHz, CDCl_3_): δ (ppm) 0.82 (t, 3H, J = 7.2 Hz, CH_3_), 1.18–1.25 (m, 4H, CH_2_), 1.36–1.40 (m, 2H, CH_2_), 1.58 (s, 3H, CH_3_), 1.91–2.06 (m, 2H, CH_2_), 3.61 (d, 1H, J = 1.2 Hz, CH), 6.01 (d, 1H, J = 1.2 Hz, =CH), 7.74–7.76 (m, 2H, Ar-H), 8.03–8.10 (m, 2H, Ar-H); ^13^C NMR (100 MHz, CDCl_3_): δ (ppm) 13.9, 18.8, 22.3, 25.1, 27.3, 31.4, 57.1, 58.0, 127.1, 127.6, 128.8, 133.4, 133.7, 134.2, 134.3, 159.2, 197.5, 198.0; HRMS (ESI), m/z calcd 269.1536 for C_18_H_21_O_2_ [M+H]^+^, found 269.1539.

*(2aS,8aS)-1-phenyl-2a,8a-dihydrocyclobuta[b]naphthalene-3,8-dione* **(3ba)**: Yellow solid, yield 87%, 153–155 °C; ^1^H NMR (400 MHz, CDCl_3_): δ (ppm) 4.06 (dd, 1H, J = 4.0 Hz, 1.6 Hz, CH), 4.45 (d, 1H, J = 4.0 Hz, CH), 6.49 (d, 1H, J = 0.8 Hz, =CH), 7.22–7.29 (m, 3H, Ar-H), 7.47–7.49 (m, 2H, Ar-H), 7.64–7.68 (m, 2H, Ar-H), 7.94–8.03 (m, 2H, Ar-H); ^13^C NMR (100 MHz, CDCl_3_): δ (ppm) 49.1, 52.2, 125.5 127.6, 127.8, 128.6, 129.2, 132.0, 133.7, 134.0, 134.6, 149.2, 195.6; HRMS (ESI), m/z calcd 261.0910 for C_18_H_13_O_2_ [M+H]^+^, found 261.0915.

*(2aR,8aR)-8a-chloro-1-phenyl-2a,8a-dihydrocyclobuta[b]naphthalene-3,8-dione* **(3ca)**: Faint yellow solid, yield 90%, 159–161 °C; ^1^H NMR (400 MHz, CDCl_3_): δ (ppm) 4.32 (d, 1H, J = 1.6 Hz, CH), 6.74 (d, 1H, J = 1.6 Hz, =CH), 7.35–7.40 (m, 3H, Ar-H), 7.64–7.67 (m, 2H, Ar-H), 7.74–7.82 (m, 2H, Ar-H), 8.06–8.14 (m, 2H, Ar-H); ^13^C NMR (100 MHz, CDCl_3_): δ (ppm) 61.6, 68.3, 126.2, 127.4, 128.7, 129.0, 129.2, 130.0, 132.3, 133.1, 135.0, 149.6, 190.0, 193.8; HRMS (ESI), m/z calcd 295.0520 for C_18_H_12_ClO_2_ [M+H]^+^, found 295.0525.

*(2aS,8aR)-2a,8a-dichloro-1-phenyl-2a,8a-dihydrocyclobuta[b]naphthalene-3,8-dione* **(3da)**: Faint yellow solid, yield 92%, 163–165 °C; ^1^H NMR (400 MHz, CDCl_3_): δ (ppm) 6.73 (s, 1H, =CH), 7.39–7.41 (m, 3H, Ar-H), 7.65–7.67 (m, 2H, Ar-H), 7.79–7.83 (m, 2H, Ar-H), 8.09–8.18 (m, 2H, Ar-H); ^13^C NMR (100 MHz, CDCl_3_): δ (ppm) 127.2, 127.4, 128.3, 128.4, 128.7, 128.9, 128.9, 131.2, 131.4, 131.7, 131.7, 135.4, 135.5, 152.6, 188.4, 188.6; HRMS (ESI), m/z calcd 329.0131 for C_18_H_10_Cl_2_O_2_ [M+H]^+^, found 329.0131.

The results of the X-ray diffraction analysis for compound **3am** were deposited with the Cambridge Crystallographic Data Centre (CCDC 2288747).

## 4. Conclusions

In summary, a facile and efficient visible-light-mediated [2+2] cycloaddition reaction strategy was developed for the synthesis of cyclobutane-containing DHCBNDOs under mild and clean conditions. The strategy exhibited favorable compatibilities with functional groups and afforded a library of DHCBNDOs in very good to excellent yields with excellent regioselectivity. Control experiments revealed that the visible light played an important role in the [2+2] cycloaddition reaction and clearly point to a radical pathway. Besides, DFT calculations revealed that the structures of transition state (**TS**) and the key intermediate (**IM1**) and its free energy barrier was calculated as 13.4 kcal mol^−1^. The readily accessible, low-cost and eco-friendly nature of the developed strategy offers a range of attractive applications in organic and medicinal chemistry.

## Figures and Tables

**Table 1:** Optimization of the [2+2] cycloaddition reaction conditions *^a^*.

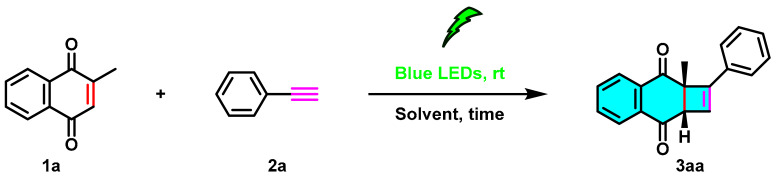				
Entry	Solvent	Time	Eqn (2a)	Yield *^b^*
1	MeCN	3 h	1.0	81%
2	DCM	3 h	1.0	59%
3	Acetone	3 h	1.0	67%
4	Dioxane	3 h	1.0	63%
5	Chlorobenzene	3 h	1.0	60%
6	MeOH	3 h	1.0	71%
7	THF	3 h	1.0	75%
8	Toluene	3 h	1.0	68%
9	ClCH_2_CH_2_Cl	3 h	1.0	64%
10	MeCN	4 h	1.0	86%
11	MeCN	5 h	1.0	85%
12	MeCN	4 h	1.1	83%
13	MeCN	4 h	1.2	84%
14	MeCN	4 h	1.4	82%
15	MeCN	4 h	1.0	0% *^c^*
16	MeCN	4 h	1.0	65% *^d^*

*^a^* The reactions were carried out on a 1 mmol scale in 10 mL of solvent under blue LEDs (460 nm); irradiation without any catalyst; *^b^* Isolated yields; *^c^* Without light; *^d^* Under irradiation of 365 nm.

## Data Availability

The data presented in this study are contained in the article tables and Supplementary Materials.

## Supplementary Materials

The following supporting information can be downloaded at: https://www.mdpi.com/article/10.3390/molecules28227654/s1, Figures S1–S40: including ^1^H, ^13^C NMR and ORTEP spectra of [2+2] cycloaddition products dihydrocyclobuta[*b*]naphthalene-3,8-diones (DHCBNDOs).
