# Supplementary material for: Visible-Light-Mediated Catalyst-Free [2+2] Cycloaddition Reaction for Dihydrocyclobuta[b]naphthalene-3,8-diones Synthesis under Mild Conditions
Source: Molecules. 2023 Nov 18;28(22):7654. doi: 10.3390/molecules28227654 (PMC10675681; doi:10.3390/molecules28227654)
Supplement: Supplementary file 1 [file molecules-28-07654-s001.zip › molecules-2713123-supplementary.pdf]

Supporting Information

Visible light-mediated [2+2] cycloaddition reaction for dihydrocyclobuta[*b*]naphthalene-3,8-diones synthesis under mild conditions

Hong-bo Tan,*[a,b] Jia-ying Zhou [a], Ying-shan Liu [a], Tong Lei [a], Shi-yu Wang [a], Shuang-shuang Hu [a], Xu Zhang [a], Zhi-gang Xu [a], Dian-yong Tang [a], Zhong-zhu Chen*[a], and Bo-chu Wang*[b]

1 National & Local Joint Engineering Research Center of Targeted and Innovative Therapeutics, Chongqing Engineering Laboratory of Targeted and Innovative Therapeutics, Chongqing Key Laboratory of Kinase Modulators as Innovative Medicine, Chongqing Collaborative Innovation Center of Targeted and Innovative Therapeutics, College of Pharmacy & IATTI, Chongqing University of Arts and Sciences, Chongqing 402160, China.

2 Key Laboratory of Biorheological Science and Technology (Chongqing University), Ministry of Education, College of Bioengineering, Chongqing University, Chongqing 400030, China.

* Correspondence: 13167859296@163.com (Hong-bo Tan); 18883138277@163.com (Zhong-zhu Chen); wangbc2000@126.com (Bo-chu Wang)

Content **Page #**

1. General procedure for preparing DHCBNDOs 1

2. Structure characterization spectra of DHCBNDOs 8

3. Cartesian coordination of stationary points 28

1. General procedure for preparing DHCBNDOs

In a 15 mL tube, 1,4-naphthoquinone **1** (1.0 mmol) and alkyne **2** (1.0 mmol) were dissolved in 10 mL of acetonitrile. The reaction mixture was under irradiation of visible blue LEDs (460 nm) for 4 h. After completion (by TLC), the reaction mixture was evaporated to dryness in a vacuo. The residue was purified by medium-pressure chromatography (silica gel) using a mixed solvent of hexane and ethyl acetate (5–30% EA).

(2aS,8aS)-8a-methyl-1-phenyl-2a,8a-dihydrocyclobuta[b]naphthalene-3,8-dione (3aa)*:* Yellow solid, yield 86%, m.p. 154-156 ℃; ^{1}H NMR (400 MHz, $CDCl_3$): δ (ppm) 1.83 (s, 3H, C*H*$_3$), 3.78 (d, 1H, *J*=1.2 Hz, C*H*), 6.55 (d, 1H, *J*=2.0 Hz, =C*H*$_2$), 7.27-7.34 (m, 3H, Ar-*H*), 7.49-7.51 (m, 2H, Ar-*H*), 7.68-7.71 (m, 2H, Ar-*H*), 8.01-8.06 (m, 2H, Ar-*H*); ^{13}C NMR (100 MHz, $CDCl_3$): δ (ppm) 19.8, 57.2, 57.5, 125.5, 127.0, 127.6, 128.0, 128.6, 129.1, 131.6, 133.7, 133.7, 134.4, 134.5, 153.4, 196.6, 198.4; HRMS (ESI), m/z calcd 275.1067 for $C_{19}H_{15}O_2$ $[M+H]^+$, found 275.1069.

(2aS,8aS)-1-(2-methoxyphenyl)-8a-methyl-2a,8a-dihydrocyclobuta[b]naphthalene-3,8-dione (3ab)*:* Faint yellow solid, yield 85%, 152-154 ℃; ^{1}H NMR (400 MHz, $CDCl_3$): δ (ppm) 1.74 (s, 3H, C*H*$_3$), 3.69 (s, 3H, OC*H*$_3$), 3.72 (d, 1H, *J*=1.6 Hz, C*H*), 6.54 (d, 1H, *J*=1.6 Hz, =C*H*$_2$), 6.72 (d, 1H, *J*=8.0 Hz, Ar-*H*), 6.85-6.88 (m, 1H, Ar-H), 7.12-7.16 (m, 1H, Ar-*H*), 7.48-7.59 (m, 3H, Ar-*H*); ^{13}C NMR (100 MHz, $CDCl_3$): δ (ppm) 20.0, 55.0, 57.5, 59.0, 110.4, 120.5, 120.6, 126.9, 127.8, 127.9, 127.9, 129.8, 132.9, 133.7, 134.0, 134.2, 134.3, 149.9, 158.9, 196.9, 199.0; HRMS (ESI), m/z calcd 305.1172 for $C_{20}H_{17}O_3$ $[M+H]^+$, found 305.1176.

***(2aS,8aS)-1-(3,5-dimethoxyphenyl)-8a-methyl-2a,8a-dihydrocyclobuta[b]naphthalene-3,8-dione (3ac)**:* Faint yellow solid, yield 88%, 151-153 ℃; ^{1}H NMR (400 MHz, $CDCl_3$): δ (ppm) 1.74 (s, 3H, C*H*$_3$), 3.68 (d, 1H, *J*=1.6 Hz, C*H*), 3.70 (s, 6H, OC*H*$_3$), 6.31 (t, 1H, *J*=2.0 Hz, Ar-*H*), 6.46 (d, 1H, *J*=1.6 Hz, =C*H*$_2$), 6.58 (t, 2H, *J*=2.0 Hz, Ar-*H*), 7.61-7.64 (m, 2H, Ar-*H*), 7.93-7.99 (m, 2H, Ar-*H*); ^{13}C NMR (100 MHz, $CDCl_3$): δ (ppm) 19.9, 55.4, 57.1, 57.4, 101.6, 103.4, 127.0, 127.6, 128.0, 128.1, 133.2, 133.6, 133.7, 134.4, 134.5, 153.3, 161.0, 196.6, 198.3; HRMS (ESI), m/z calcd 335.1278 for $C_{21}H_{19}O_4$ $[M+H]^+$, found 335.1280.

***(2aS,8aS)-1-(4-ethylphenyl)-8a-methyl-2a,8a-dihydrocyclobuta[b]naphthalene-3,8-dione (3ad)**:* Yellow solid, yield 93%, 155-157 ℃; ^{1}H NMR (400 MHz, $CDCl_3$): δ (ppm) 1.20 (t, 3H, *J*=7.6 Hz, C*H*$_3$), 1.83 (s, 3H, C*H*$_3$), 2.61 (q, 2H, *J*=7.6 Hz, C*H*$_2$), 3.77 (d, 1H, *J*=1.6 Hz, C*H*), 6.49 (d, 1H, *J*=1.6 Hz, =C*H*$_2$), 7.16 (d, 2H, *J*=8.4 Hz, Ar-*H*), 7.42 (d, 2H, *J*=8.4 Hz, Ar-*H*), 7.69-7.72 (m, 2H, Ar-*H*), 8.02-8.06 (m, 2H, Ar-*H*); ^{13}C NMR (100 MHz, $CDCl_3$): δ (ppm) 15.4, 19.8, 28.8, 57.1, 57.5, 125.6, 126.4, 127.0, 128.0, 128.1, 129.2, 133.7, 133.8, 134.3, 134.4, 145.6, 153.5, 196.9, 198.6; HRMS (ESI), m/z calcd 303.1380 for $C_{21}H_{19}O_2$ $[M+H]^+$, found 303.1385.

***(2aS,8aS)-8a-methyl-1-(4-propylphenyl)-2a,8a-dihydrocyclobuta[b]naphthalene-3,8-dione (3ae)**:* Yellow solid, yield 90%, 154-156 ℃; ^{1}H NMR (400 MHz, $CDCl_3$): δ (ppm) 0.84 (t, 3H, *J*=7.2 Hz, C*H*$_3$), 1.53 (q, 2H, *J*=7.6 Hz, C*H*$_2$), 1.75 (s, 3H, C*H*$_3$), 2.47 (t, 2H, C*H*$_2$), 3.70 (d, 1H, *J*=1.6 Hz, C*H*), 6.40 (d, 1H, *J*=1.6 Hz, =C*H*$_2$), 7.06 (d, 2H, *J*=8.0 Hz, Ar-*H*), 7.34 (d, 2H, *J*=8.4 Hz, Ar-*H*), 7.62-7.65 (m, 2H, Ar-*H*), 7.94-7.99 (m, 2H, Ar-*H*); ^{13}C NMR (100 MHz, $CDCl_3$): δ (ppm) 13.8, 19.8, 24.4, 37.9, 57.1, 57.5, 125.5, 126.4, 127.0, 128.0, 128.7, 129.2, 133.7, 133.8, 134.3, 134.4, 144.0, 153.5, 196.9, 198.6; HRMS (ESI), m/z calcd 317.1536 for $C_{22}H_{21}O_2$ $[M+H]^+$, found 317.1539.

(2aS,8aS)-8a-methyl-1-(4-((1s,4S)-4-propylcyclohexyl)phenyl)-2a,8a-dihydrocyclobuta[b]naphthalene-3,8-dione (3af):Yellow solid, yield 95%, 149-151 ℃; ^{1}H NMR (400 MHz, $CDCl_3$): δ (ppm) 0.89 (t, 3H, *J*=7.2 Hz, CH_3), 1.04-1.07 (m, 2H, CH_2), 1.18-1.22 (m, 2H, CH_2), 1.28-1.42 (m, 6H, CH_2), 1.82 (s, 3H, CH_3), 1.85 (s, 3H, CH_3), 2.40-2.46 (m, 1H, C*H*), 3.76 (d, 1H, *J*=1.6 Hz, C*H*), 6.48 (d, 1H, *J*=1.6 Hz, =CH_2), 7.17 (d, 2H, *J*=8.4 Hz, Ar-*H*), 7.42 (d, 2H, *J*=8.4 Hz, Ar-*H*), 7.68-7.72 (m, 2H, Ar-*H*), 8.01-8.06 (m, 2H, Ar-*H*); ^{13}C NMR (100 MHz, $CDCl_3$): δ (ppm) 14.4, 19.9, 20.0, 33.5, 34.1, 37.0, 39.7, 44.6, 57.1, 57.5, 125.5, 126.4, 127.0, 128.0, 129.3, 133.7, 133.7, 134.3, 134.4, 149.2, 153.5, 196.8, 198.5; HRMS (ESI), m/z calcd 399.2319 for $C_{28}H_{31}O_2$ $[M+H]^+$, found 399.2323.

(2aS,8aS)-1-(4-bromophenyl)-8a-methyl-2a,8a-dihydrocyclobuta[b]naphthalene-3,8-dione (3ag): Yellow solid, yield 83%, 165-167 ℃; ^{1}H NMR (400 MHz, $CDCl_3$): δ (ppm) 1.72 (s, 3H, CH_3), 3.68 (d, 1H, *J*=1.6 Hz, C*H*), 6.48 (d, 1H, *J*=2.0 Hz, =CH_2), 7.28 (d, 2H, *J*=8.8 Hz, Ar-*H*), 7.36 (d, 2H, *J*=8.8 Hz, Ar-*H*), 7.62-7.65 (m, 2H, Ar-*H*), 7.94-7.98 (m, 2H, Ar-*H*); ^{13}C NMR (100 MHz, $CDCl_3$): δ (ppm) 19.8, 57.1, 57.5, 123.3, 127.1, 127.1, 128.0, 128.3, 130.3, 131.9, 133.5, 133.6, 134.5, 134.6, 152.2, 196.2, 198.2; HRMS (ESI), m/z calcd 353.0172 for $C_{19}H_{14}BrO_2$ $[M+H]^+$, found 353.0176.

(2aS,8aS)-8a-methyl-1-(4-nitrophenyl)-2a,8a-dihydrocyclobuta[b]naphthalene-3,8-dione (3ah): Yellow solid, yield 80%, 173-175 ℃; ^{1}H NMR (400 MHz, $CDCl_3$): δ (ppm) 1.78 (s, 3H, CH_3), 3.78 (d, 1H, *J*=1.6 Hz, C*H*), 6.72 (d, 1H, *J*=2.0 Hz, =CH_2), 7.62 (d, 2H, *J*=8.8 Hz, Ar-*H*), 7.68-7.71 (m, 2H, Ar-*H*), 7.99-8.03 (m, 2H, Ar-*H*), 8.11 (d, 2H, *J*=8.8 Hz, Ar-*H*); ^{13}C NMR (100 MHz, $CDCl_3$): δ (ppm) 19.9, 57.2, 57.7, 124.0, 126.4, 127.2, 128.2, 133.4, 133.6, 134.8, 134.8,

137.1, 147.6, 151.1, 195.4, 197.6; HRMS (ESI), m/z calcd 320.0917 for $C_{19}H_{14}NO_4$ $[M+H]^+$, found 320.0921.

4-((2aS,8aS)-8a-methyl-3,8-dioxo-2a,3,8,8a-tetrahydrocyclobuta[b]naphthalen-1-yl)benzonitrile (3ai): Yellow solid, yield 82%, 170-172 ℃; ^{1}H NMR (400 MHz, $CDCl_3$): δ (ppm) 1.75 (s, 3H, CH_3), 3.75 (br s, 1H, C*H*), 6.66 (br s, 1H, =CH_2), 7.54 (br s, 4H, Ar-*H*), 7.66-7.68 (m, 2H, Ar-*H*), 7.96-8.01 (m, 2H, Ar-*H*); ^{13}C NMR (100 MHz, $CDCl_3$): δ (ppm) 19.9, 57.2, 57.6, 112.3, 118.5, 126.1, 127.2, 128.1, 131.7, 132.4, 133.4, 133.6, 134.7, 134.7, 135.3, 151.4, 195.5, 197.7; HRMS (ESI), m/z calcd 300.1019 for $C_{20}H_{14}NO_2$ $[M+H]^+$, found 300.1022.

(2aS,8aS)-8a-methyl-1-(pyridin-3-yl)-2a,8a-dihydrocyclobuta[b]naphthalene-3,8-dione (3aj): Yellow solid, yield 93%, 168-170 ℃; ^{1}H NMR (400 MHz, $CDCl_3$): δ (ppm) 1.74 (s, 3H, CH_3), 3.74 (d, 1H, *J*=1.6 Hz, C*H*), 6.59 (d, 1H, *J*=2.0 Hz, =CH_2), 6.17 (dd, 1H, *J*=8.0 Hz, 4.8 Hz, Ar-*H*), 7.63-7.66 (m, 2H, Ar-*H*), 7.74 (dt, 1H, *J*=8.0 Hz, 2.0 Hz, Ar-*H*), 7.94-7.99 (m, 2H, Ar-*H*), 8.39 (dd, 1H, *J*=4.8 Hz, 1.6 Hz, Ar-*H*), 8.65 (d, 1H, *J*=2.0 Hz, Ar-*H*); ^{13}C NMR (100 MHz, $CDCl_3$): δ (ppm) 19.8, 57.2, 57.7, 123.5, 127.1, 127.4, 128.0, 129.9, 132.7, 133.6, 134.6, 134.6, 147.0, 149.6, 150.5, 195.9, 197.8; HRMS (ESI), m/z calcd 276.1019 for $C_{18}H_{14}NO_2$ $[M+H]^+$, found 276.1024.

(2aS,8aS)-8a-methyl-1-(thiophen-2-yl)-2a,8a-dihydrocyclobuta[b]naphthalene-3,8-dione (3ak): Faint yellow solid, yield 91%, 164-166 ℃; ^{1}H NMR (400 MHz, $CDCl_3$): δ (ppm) 1.72 (s, 3H, CH_3), 3.71 (d, 1H, *J*=1.6 Hz, C*H*), 6.17 (d, 1H, *J*=1.6 Hz, =CH_2), 6.90 (dd, 1H, *J*=4.8 Hz, 3.6 Hz, Ar-*H*), 7.18-7.20 (m, 2H, Ar-*H*), 7.61-7.64 (m, 2H, Ar-*H*), 7.94-7.99 (m, 2H, Ar-*H*); ^{13}C NMR (100 MHz, $CDCl_3$): δ (ppm) 19.7, 57.5, 57.8, 125.0, 126.7, 126.8, 127.2, 127.7, 128.0, 133.4,

133.8, 134.4, 134.5, 134.5, 147.6, 196.2, 197.5; HRMS (ESI), m/z calcd 281.0631 for $C_{17}H_{13}O_2S$ $[M+H]^+$, found 281.0635.

(2aS,8aS)-2a-methyl-1,2-diphenyl-2a,8a-dihydrocyclobuta[b]naphthalene-3,8-dione (3al): Yellow solid, yield 83%, 160-162 ℃; ^1H NMR (400 MHz, $CDCl_3$): δ (ppm) 1.80 (s, 3H, C*H*3), 4.21 (s, 1H, C*H*), 7.28-7.37 (m, 6H, Ar-*H*), 7.51-7.55 (m, 4H, Ar-*H*), 7.68-7.77 (m, 2H, Ar-*H*), 7.93 (dd, 1H, *J*=7.6 Hz, 1.2 Hz, Ar-*H*), 8.13 (dd, 1H, *J*=7.6 Hz, 1.2 Hz, Ar-*H*); ^{13}C NMR (100 MHz, $CDCl_3$): δ (ppm) 19.3, 55.5, 58.5, 126.9, 127.0, 127.0, 127.9, 128.5, 128.7, 128.9, 128.9, 132.8, 132.8, 133.9, 134.0, 134.3, 134.5, 140.6, 145.0, 196.7, 198.6; HRMS (ESI), m/z calcd 351.1380 for $C_{25}H_{19}O_2$ $[M+H]^+$, found 351.1385.

(2aS,8aS)-2a-methyl-3,8-dioxo-2-phenyl-2a,3,8,8a-tetrahydrocyclobuta[b]naphthalene-1-carbaldehyde (3am): Faint yellow solid, yield 85%, 173-175 ℃; ^1H NMR (400 MHz, $CDCl_3$): δ (ppm) 1.81 (s, 3H, C*H*3), 4.00 (s, 1H, C*H*), 7.33-7.39 (m, 3H, Ar-*H*), 7.63-7.68 (m, 2H, Ar-*H*), 7.84 (dd, 2H, *J*=8.0 Hz, 2.0 Hz, Ar-*H*), 7.94 (d, 1H, *J*=7.6 Hz, Ar-*H*), 9.85 (s, 1H, C*H*O); ^{13}C NMR (100 MHz, $CDCl_3$): δ (ppm) 20.3, 56.2, 127.4, 128.0, 129.1, 129.3, 130.8, 132.0, 133.5, 133.8, 134.6, 134.9, 135.0, 161.1, 185.0, 194.4, 196.7; HRMS (ESI), m/z calcd 303.1016 for $C_{20}H_{15}O_3$ $[M+H]^+$, found 303.1020.

(2aS,8aS)-1-butyl-8a-methyl-2a,8a-dihydrocyclobuta[b]naphthalene-3,8-dione (3an): Faint yellow oil, yield 95%; ^1H NMR (400 MHz, $CDCl_3$): δ (ppm) 0.83 (t, 3H, *J*=7.2 Hz, C*H*3), 1.21-1.39 (m, 4H, C*H*2), 1.59 (s, 3H, C*H*3), 1.92-2.08 (m, 2H, C*H*2), 3.62 (d, 1H, *J*=1.2 Hz, C*H*), 6.01 (d, 1H, *J*=1.2 Hz, =C*H*2), 7.74-7.76 (m, 2H, Ar-*H*), 8.03-8.10 (m, 2H, Ar-*H*); ^{13}C NMR (100 MHz, $CDCl_3$): δ (ppm) 13.7, 18.9, 22.3, 27.0, 27.5, 57.1, 58.0, 127.1, 127.6, 128.8, 133.4,

133.8, 134.3, 134.3, 159.1, 197.6, 198.1; HRMS (ESI), m/z calcd 255.1380 for $C_{17}H_{19}O_2$ $[M+H]^+$, found 255.1385.

(2aS,8aS)-8a-methyl-1-pentyl-2a,8a-dihydrocyclobuta[b]naphthalene-3,8-dione (3ao): Faint yellow oil, yield 96%; ^{1}H NMR (400 MHz, $CDCl_3$): δ (ppm) 0.82 (t, 3H, *J*=7.2 Hz, C*H*$_3$), 1.18-1.25 (m, 4H, C*H*$_2$), 1.36-1.40 (m, 2H, C*H*$_2$), 1.58 (s, 3H, C*H*$_3$), 1.91-2.06 (m, 2H, C*H*$_2$), 3.61 (d, 1H, *J*=1.2 Hz, C*H*), 6.01 (d, 1H, *J*=1.2 Hz, =C*H*$_2$), 7.74-7.76 (m, 2H, Ar-*H*), 8.03-8.10 (m, 2H, Ar-*H*); ^{13}C NMR (100 MHz, $CDCl_3$): δ (ppm) 13.9, 18.8, 22.3, 25.1, 27.3, 31.4, 57.1, 58.0, 127.1, 127.6, 128.8, 133.4, 133.7, 134.2, 134.3, 159.2, 197.5, 198.0; HRMS (ESI), m/z calcd 269.1536 for $C_{18}H_{21}O_2$ $[M+H]^+$, found 269.1539.

(2aS,8aS)-1-phenyl-2a,8a-dihydrocyclobuta[b]naphthalene-3,8-dione (3ba): Yellow solid, yield 87%, 153-155 ℃; ^{1}H NMR (400 MHz, $CDCl_3$): δ (ppm) 4.06 (dd, 1H, *J*=4.0 Hz, 1.6 Hz, C*H*), 4.45 (d, 1H, *J*=4.0 Hz, C*H*), 6.49 (d, 1H, *J*=0.8 Hz, =C*H*$_2$), 7.22-7.29 (m, 3H, Ar-*H*), 7.47-7.49 (m, 2H, Ar-*H*), 7.64-7.68 (m, 2H, Ar-*H*), 7.94-8.03 (m, 2H, Ar-*H*); ^{13}C NMR (100 MHz, $CDCl_3$): δ (ppm) 49.1, 52.2, 125.5 127.6, 127.8, 128.6, 129.2, 132.0, 133.7, 134.0, 134.6, 149.2, 195.6; HRMS (ESI), m/z calcd 261.0910 for $C_{18}H_{13}O_2$ $[M+H]^+$, found 261.0915.

(2aR,8aR)-8a-chloro-1-phenyl-2a,8a-dihydrocyclobuta[b]naphthalene-3,8-dione (3ca): Faint yellow solid, yield 90%, 159-161 ℃; ^{1}H NMR (400 MHz, $CDCl_3$): δ (ppm) 4.32 (d, 1H, *J*=1.6 Hz, C*H*), 6.74 (d, 1H, *J*=1.6 Hz, =C*H*$_2$), 7.35-7.40 (m, 3H, Ar-*H*), 7.64-7.67 (m, 2H, Ar-*H*), 7.74-7.82 (m, 2H, Ar-*H*), 8.06-8.14 (m, 2H, Ar-*H*); ^{13}C NMR (100 MHz, $CDCl_3$): δ (ppm) 61.6, 68.3, 126.2, 127.4, 128.7, 129.0, 129.2, 130.0, 132.3, 133.1, 135.0, 149.6, 190.0, 193.8; HRMS (ESI), m/z calcd 295.0520 for $C_{18}H_{12}ClO_2$ $[M+H]^+$, found 295.0525.

***(2aS,8aR)-2a,8a-dichloro-1-phenyl-2a,8a-dihydrocyclobuta[b]naphthalene-3,8-dione (3da)**:*

Faint yellow solid, yield 92%, 163-165 ℃; ^{1}H NMR (400 MHz, $CDCl_3$): δ (ppm) 6.73 (s, 1H, =CH_2), 7.39-7.41 (m, 3H, Ar-*H*), 7.65-7.67 (m, 2H, Ar-*H*), 7.79-7.83 (m, 2H, Ar-*H*), 8.09-8.18 (m, 2H, Ar-*H*); ^{13}C NMR (100 MHz, $CDCl_3$): δ (ppm) 127.2, 127.4, 128.3, 128.4, 128.7, 128.9, 128.9, 131.2, 131.4, 131.7, 131.7, 135.4, 135.5, 152.6, 188.4, 188.6; HRMS (ESI), m/z calcd 329.0131 for $C_{18}H_{11}ClO_2$ $[M+H]^+$, found 329.0131.

2. Structure characterization spectra of DHCBNDOs

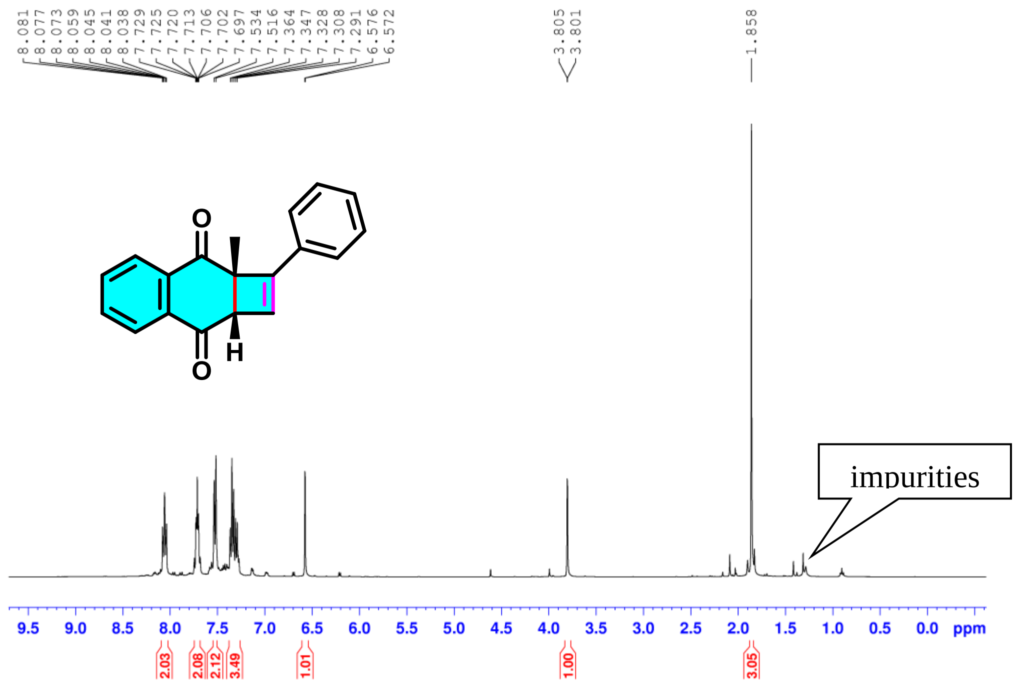

SI-Fig. 1. ^{1}H NMR spectrum of compound **3aa**.

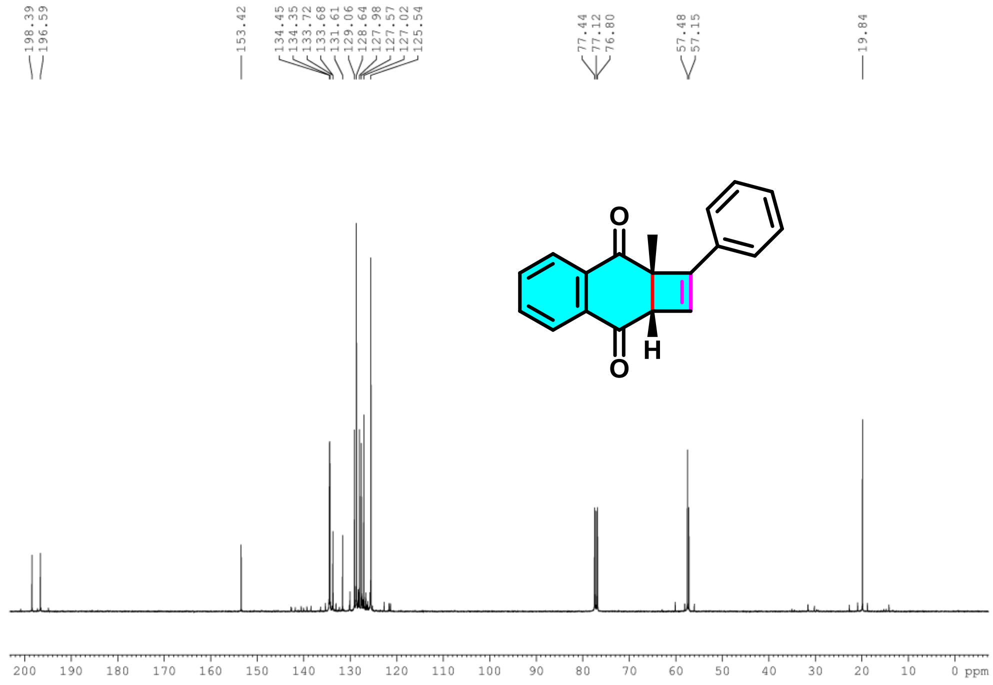

SI-Fig. 2. ^{13}C NMR spectrum of compound **3aa**.

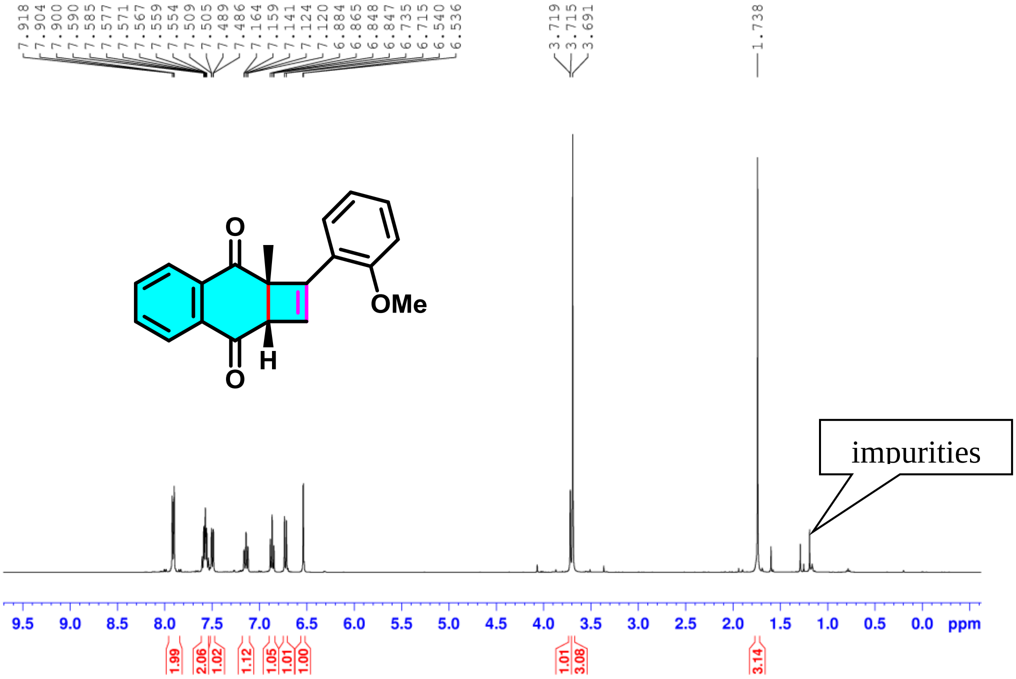

SI-Fig. 3. ^{1}H NMR spectrum of compound **3ab**.

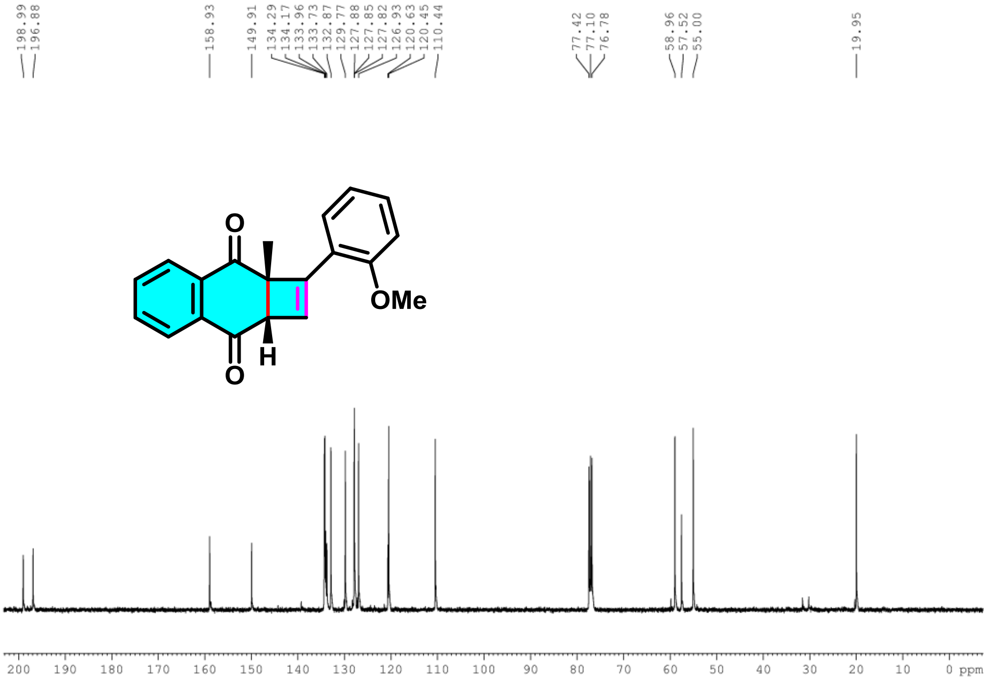

SI-Fig. 4. ^{13}C NMR spectrum of compound **3ab**.

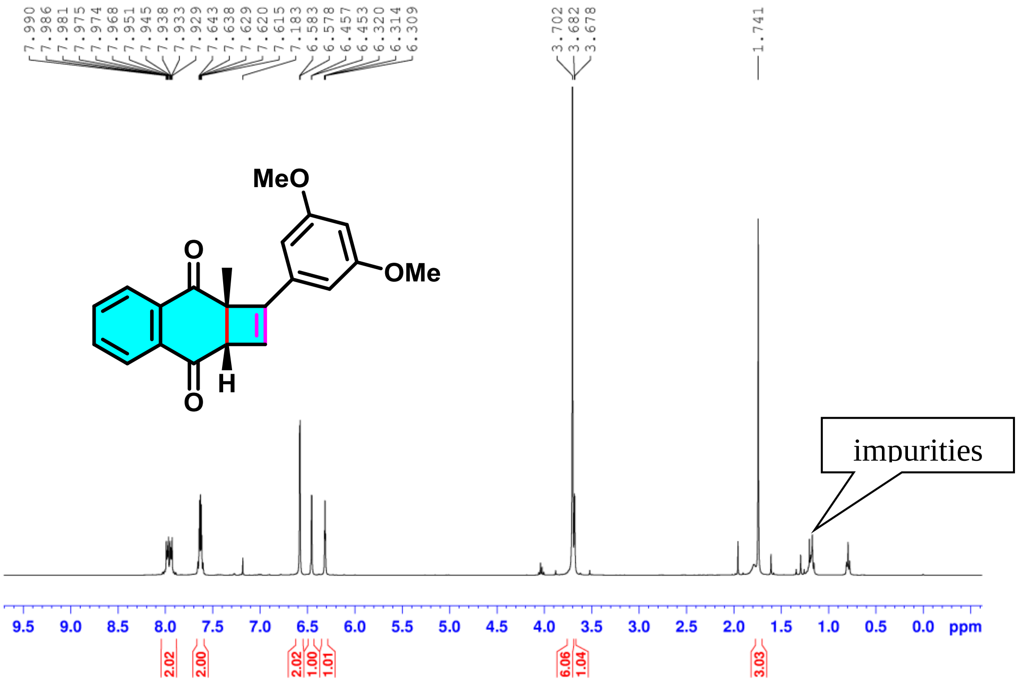

SI-Fig. 5. ^{1}H NMR spectrum of compound **3ac**.

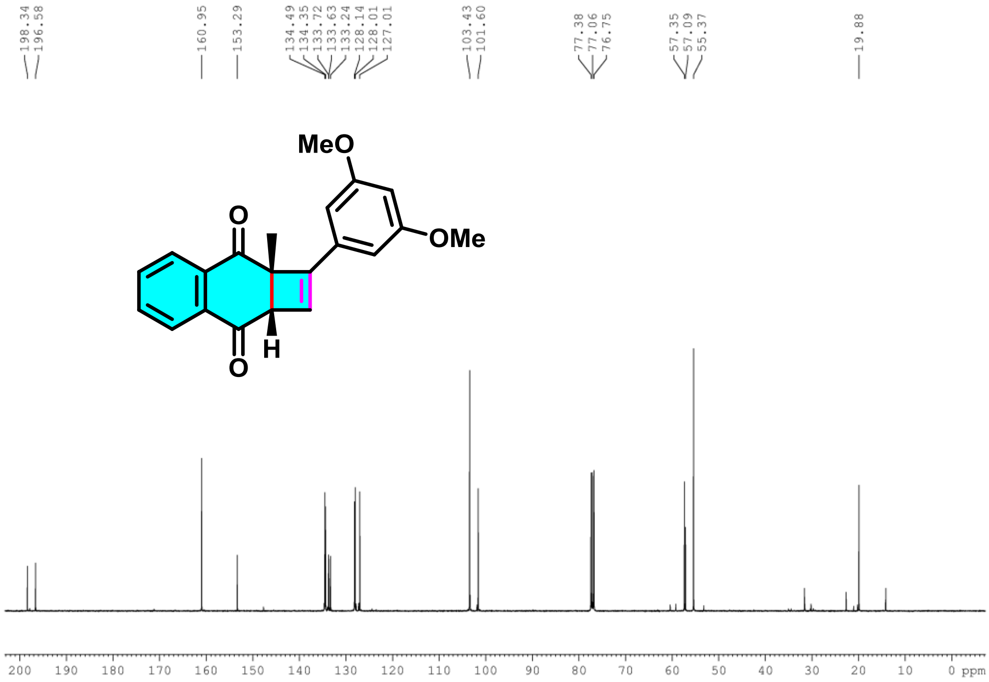

SI-Fig. 6. ^{13}C NMR spectrum of compound **3ac**.

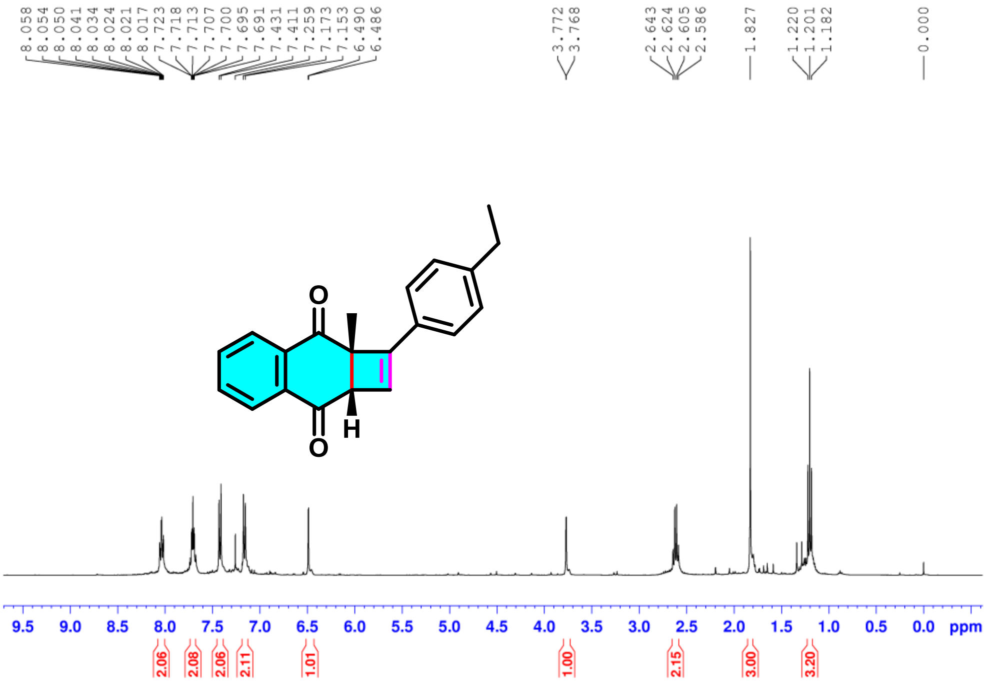

SI-Fig. 7. ^{1}H NMR spectrum of compound **3ad**.

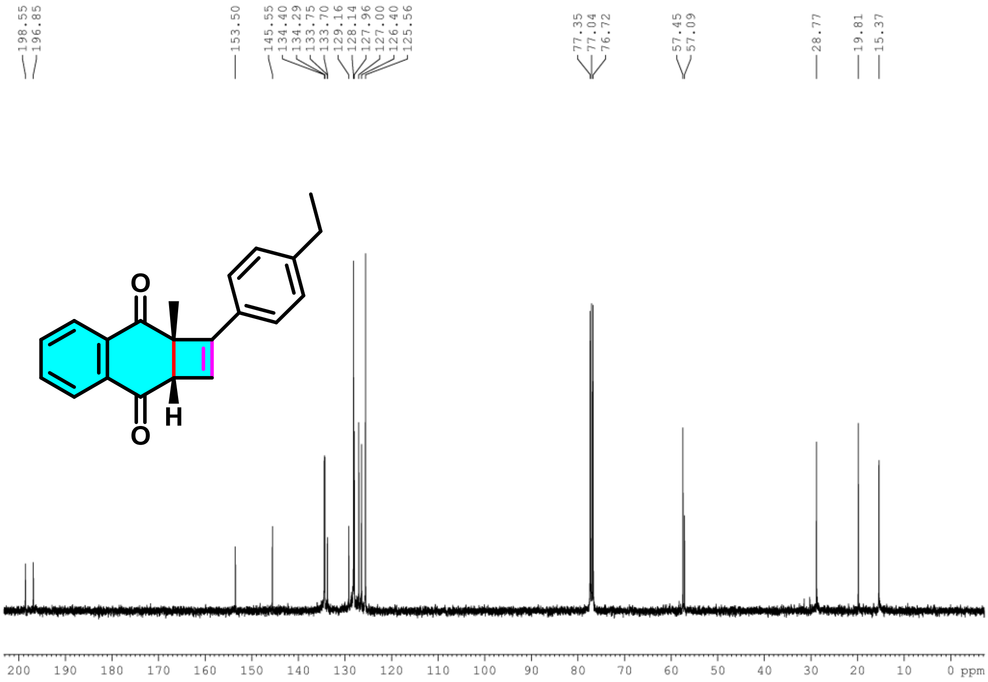

SI-Fig. 8. ^{13}C NMR spectrum of compound **3ad**.

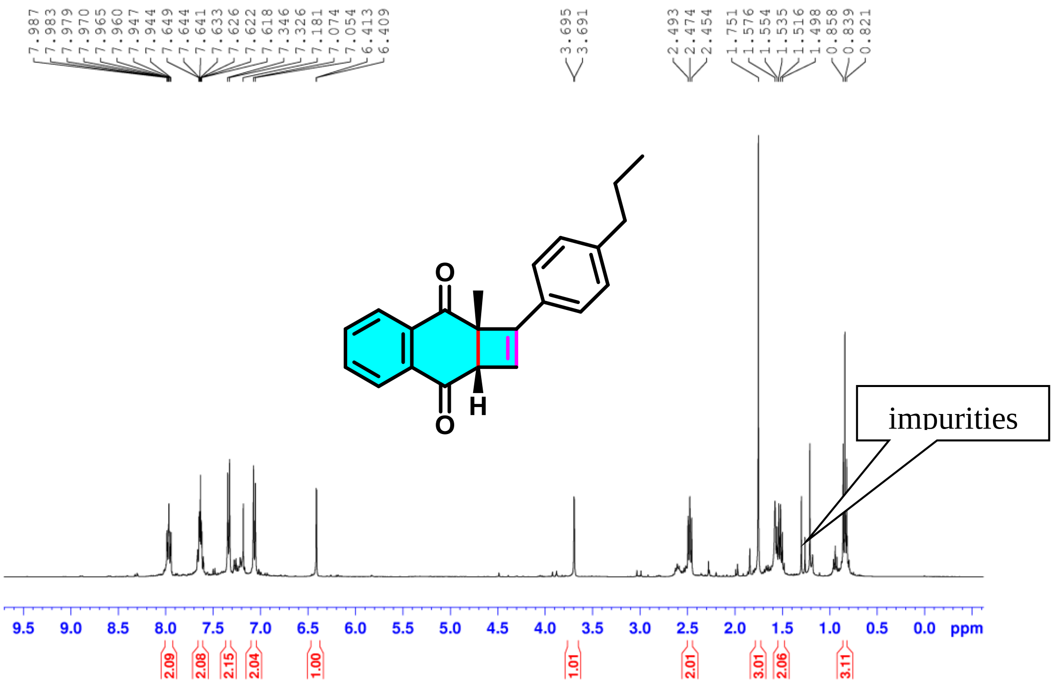

SI-Fig. 9. ^{1}H NMR spectrum of compound **3ae**.

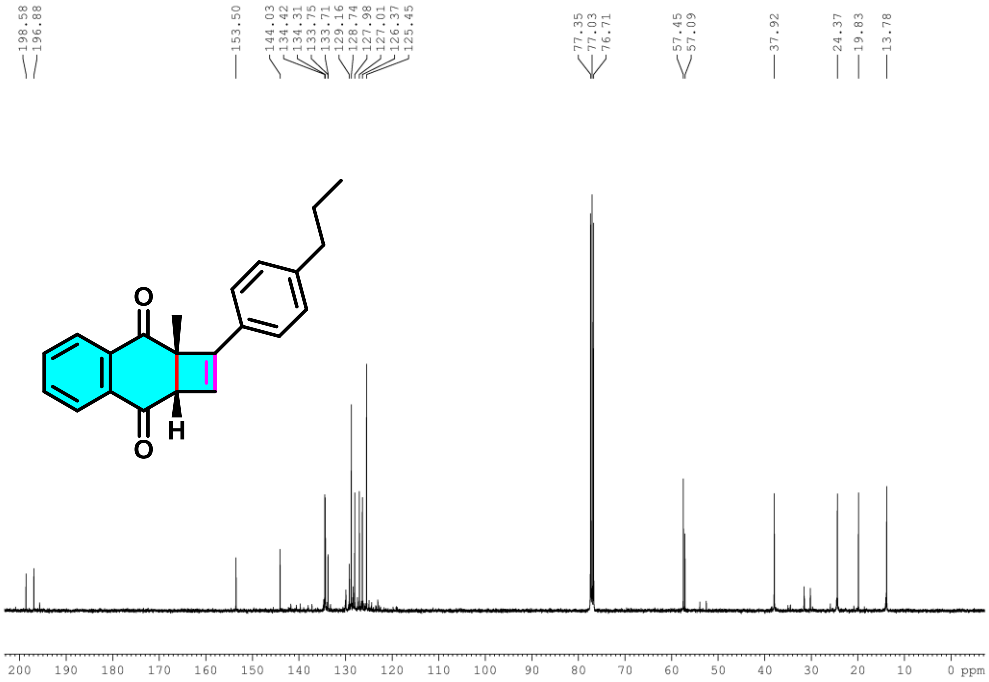

SI-Fig. 10. ^{13}C NMR spectrum of compound **3ae**.

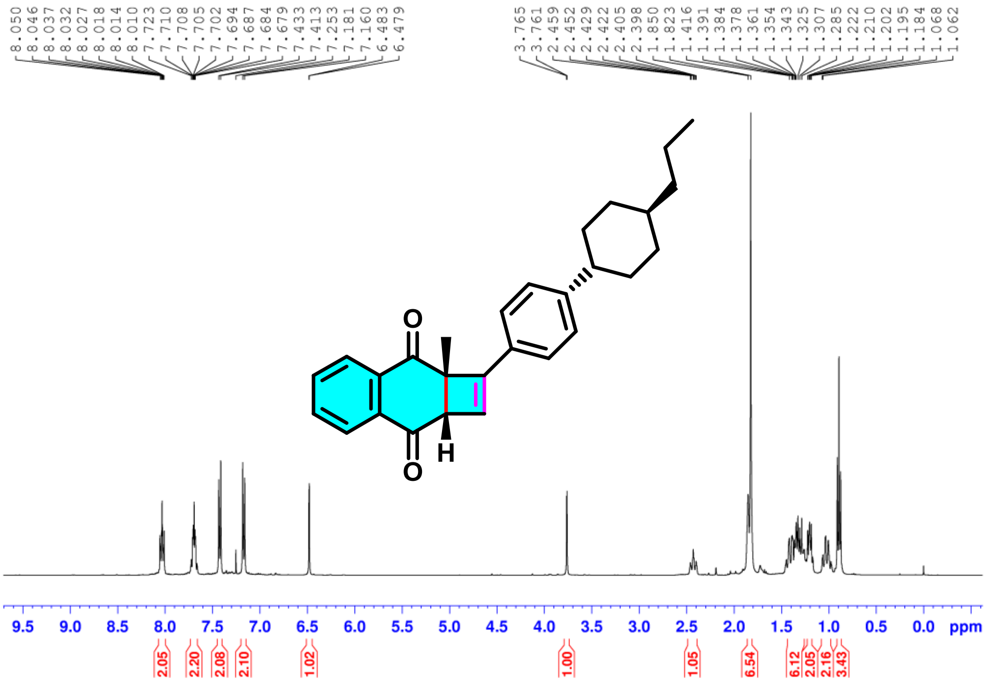

SI-Fig. 11. ^{1}H NMR spectrum of compound **3af**.

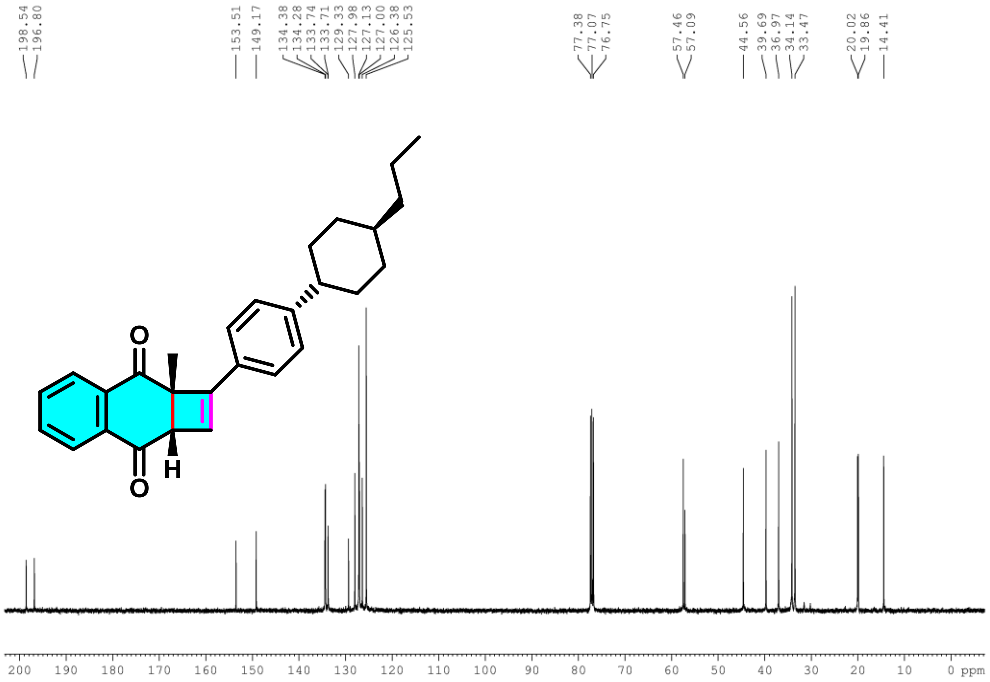

SI-Fig. 12. ^{13}C NMR spectrum of compound **3af**.

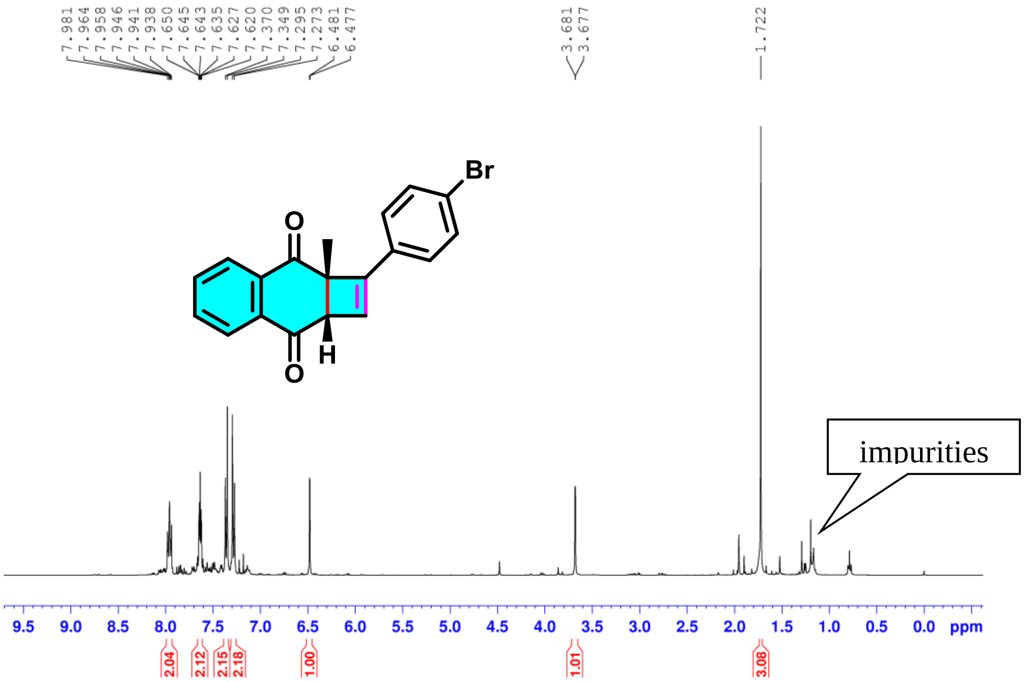

SI-Fig. 13. ^{1}H NMR spectrum of compound **3ag**.

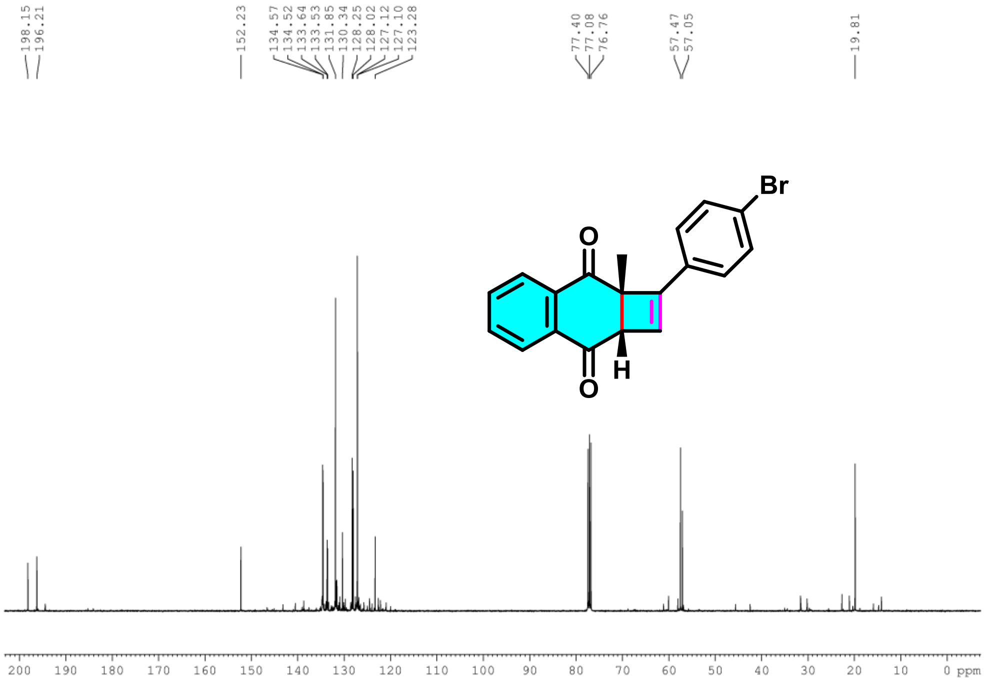

SI-Fig. 14. ^{13}C NMR spectrum of compound **3ag**.

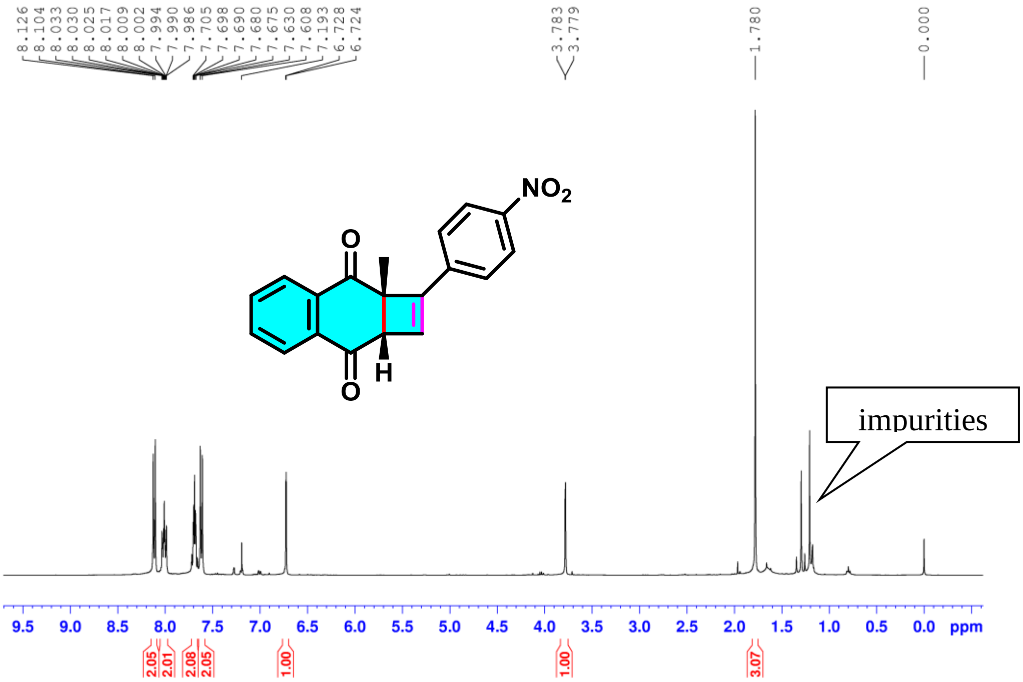

SI-Fig. 15. ^{1}H NMR spectrum of compound **3ah**.

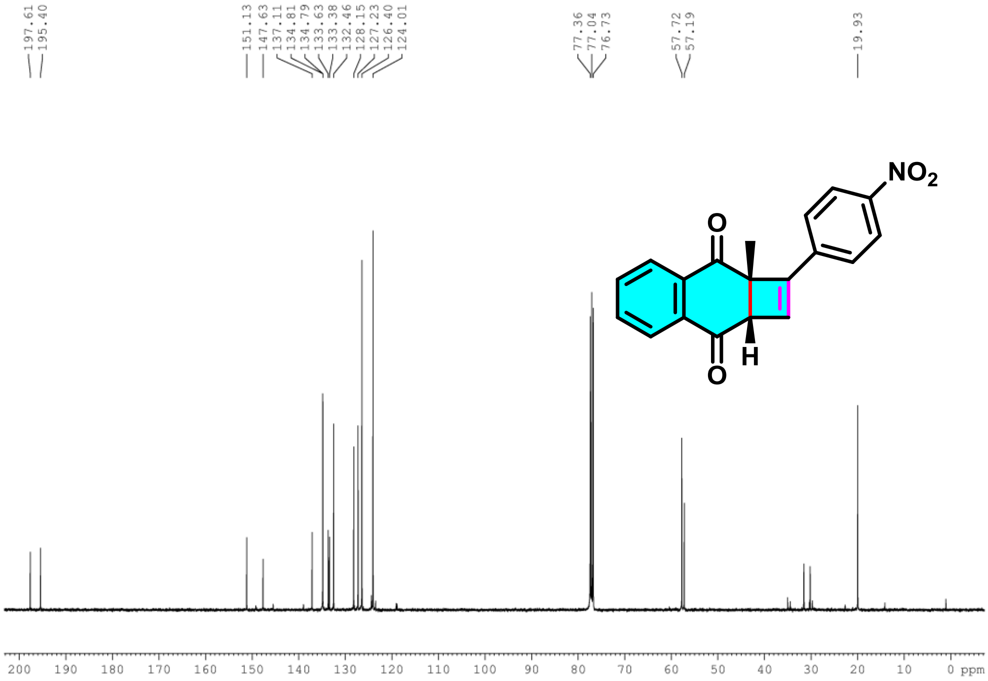

SI-Fig. 16. ^{13}C NMR spectrum of compound **3ah**.

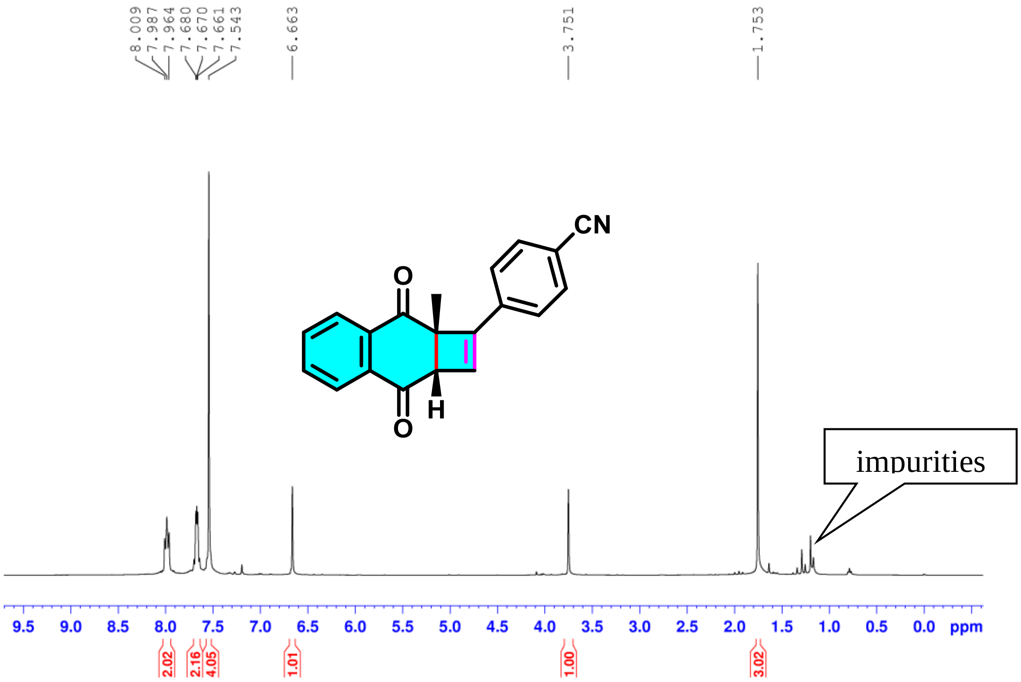

SI-Fig. 17. ^{1}H NMR spectrum of compound **3ai**.

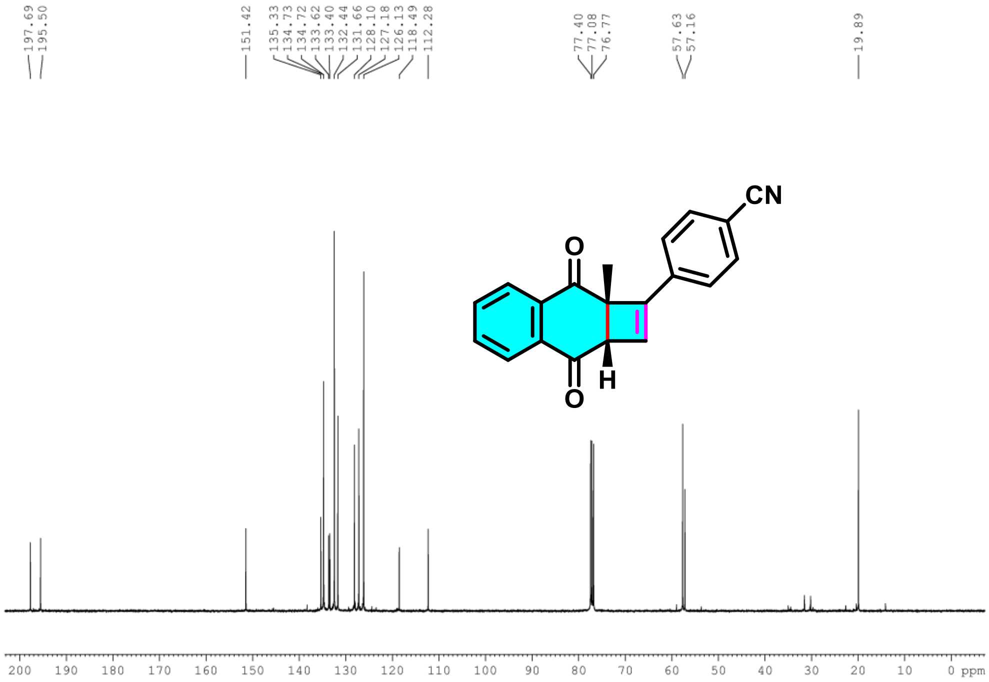

SI-Fig. 18. ^{13}C NMR spectrum of compound **3ai**.

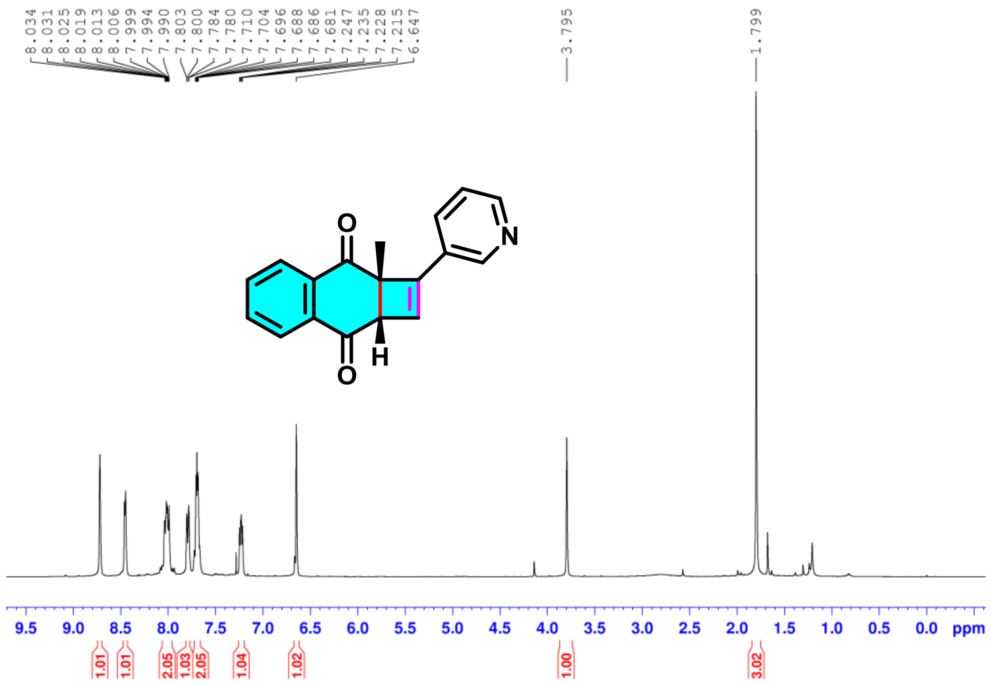

SI-Fig. 19. ^{1}H NMR spectrum of compound **3aj**.

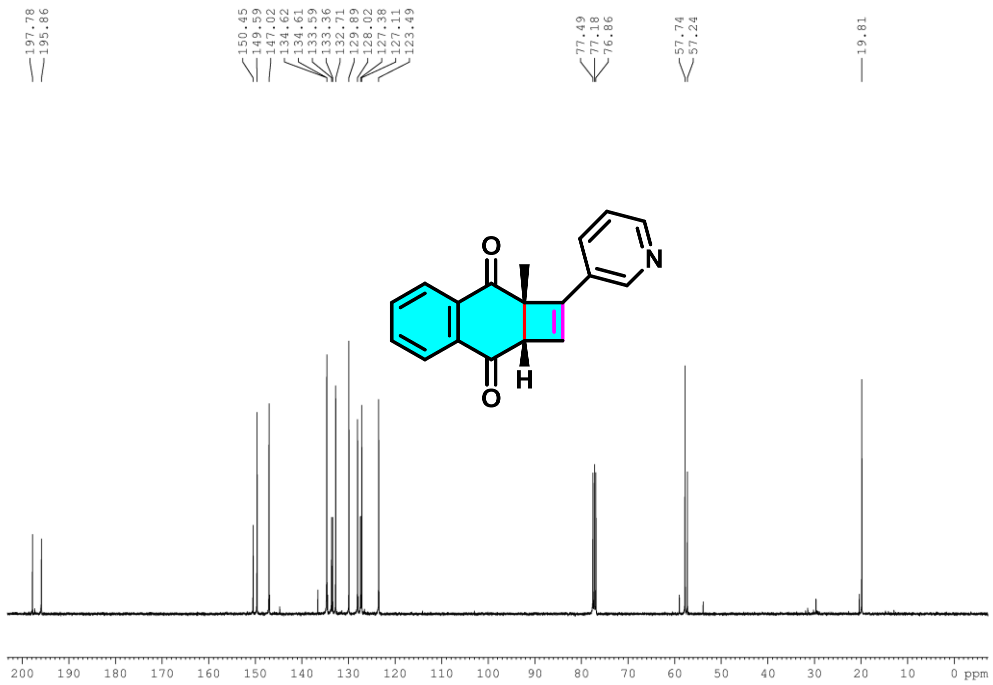

SI-Fig. 20. ^{13}C NMR spectrum of compound **3aj**.

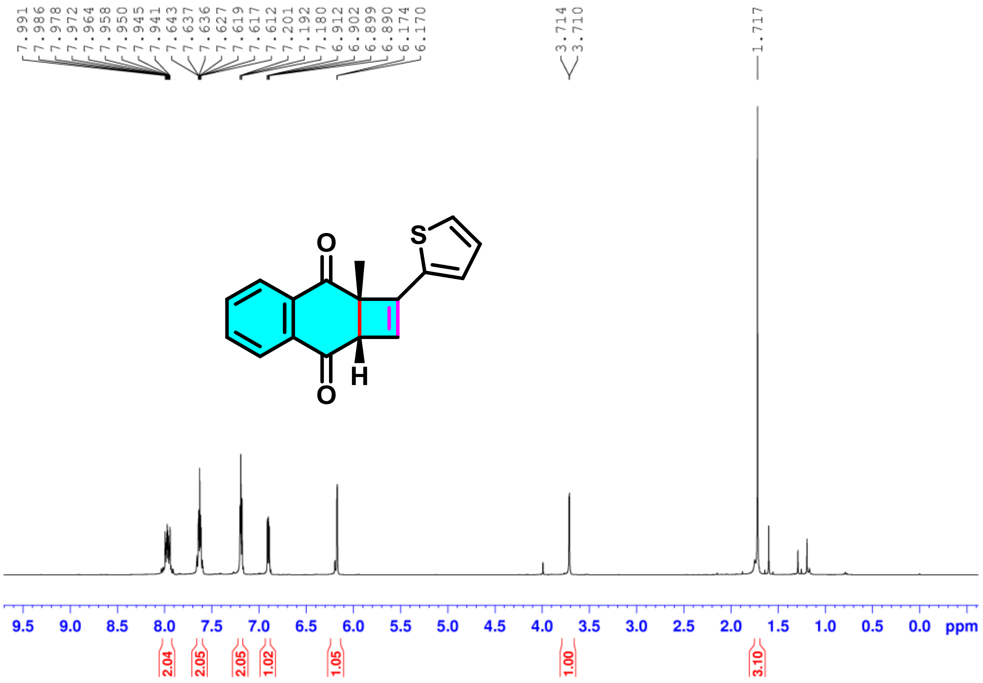

SI-Fig. 21. ^{1}H NMR spectrum of compound **3ak**.

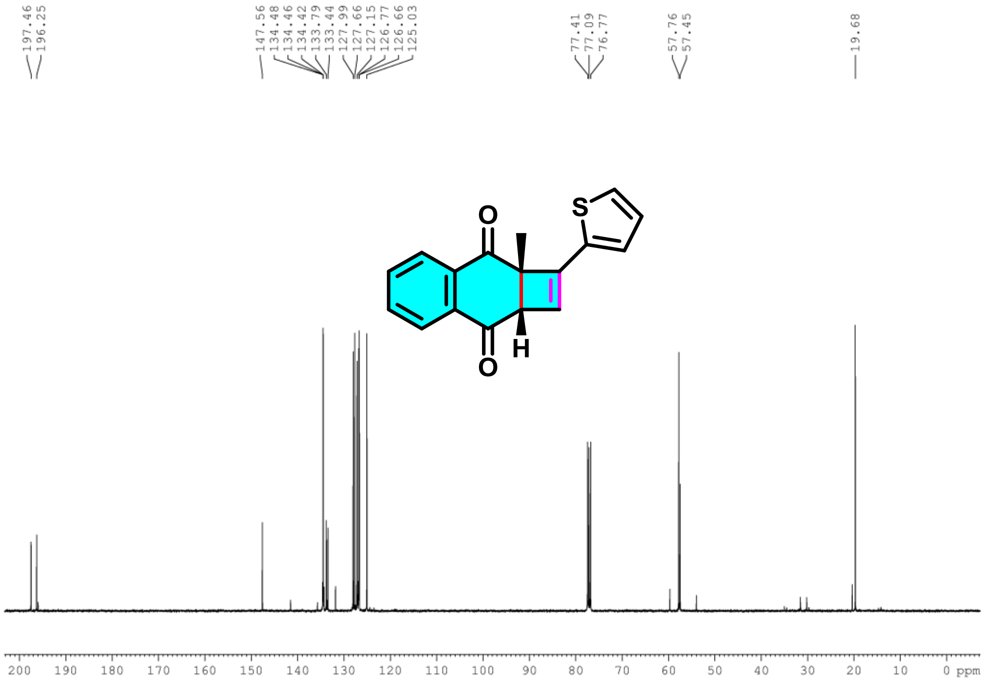

SI-Fig. 22. ^{13}C NMR spectrum of compound **3ak**.

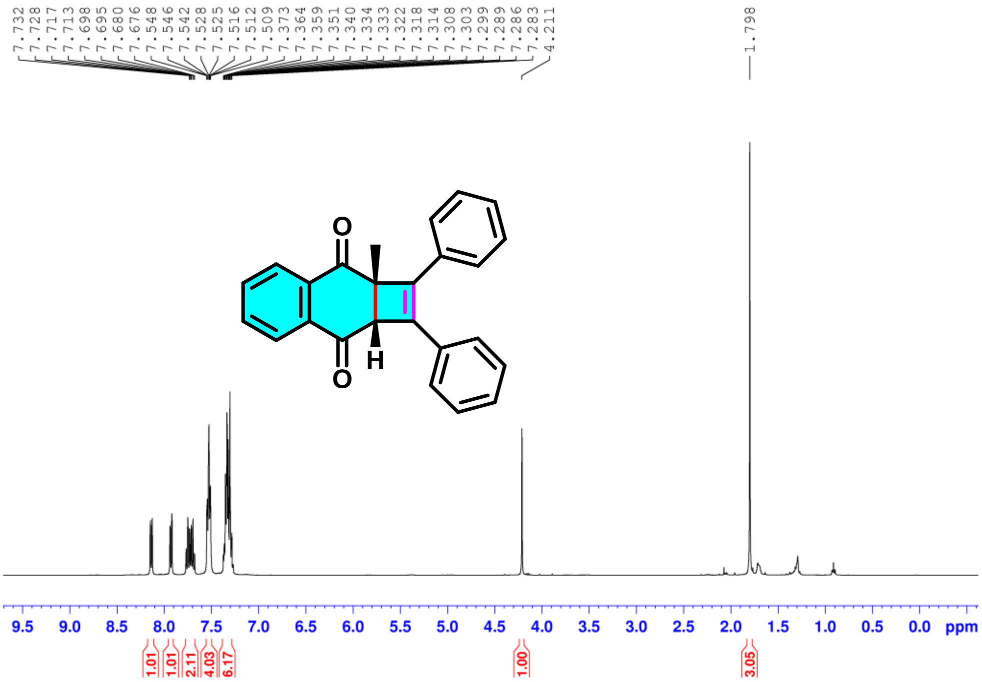

SI-Fig. 23. ^{1}H NMR spectrum of compound **3al**.

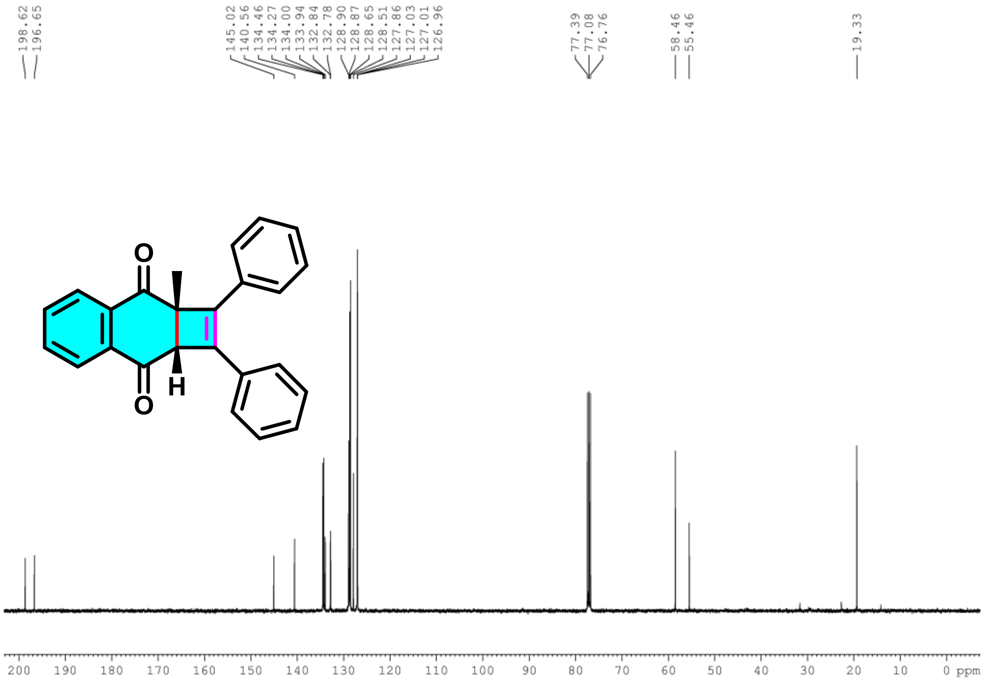

SI-Fig. 24. ^{13}C NMR spectrum of compound **3al**.

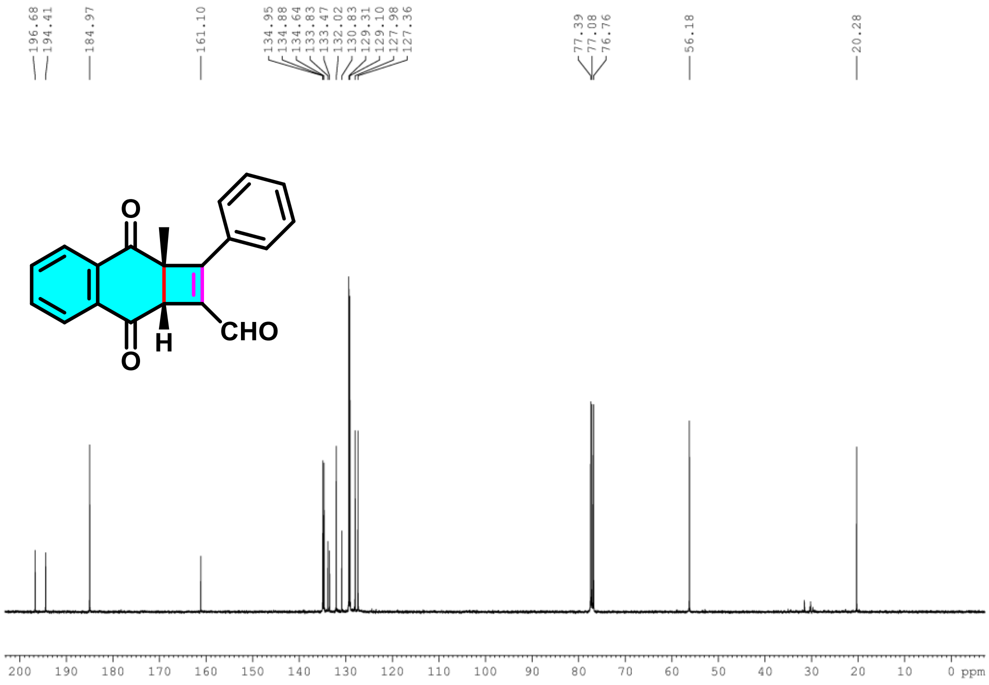

SI-Fig. 25. ^{1}H NMR spectrum of compound **3am**.

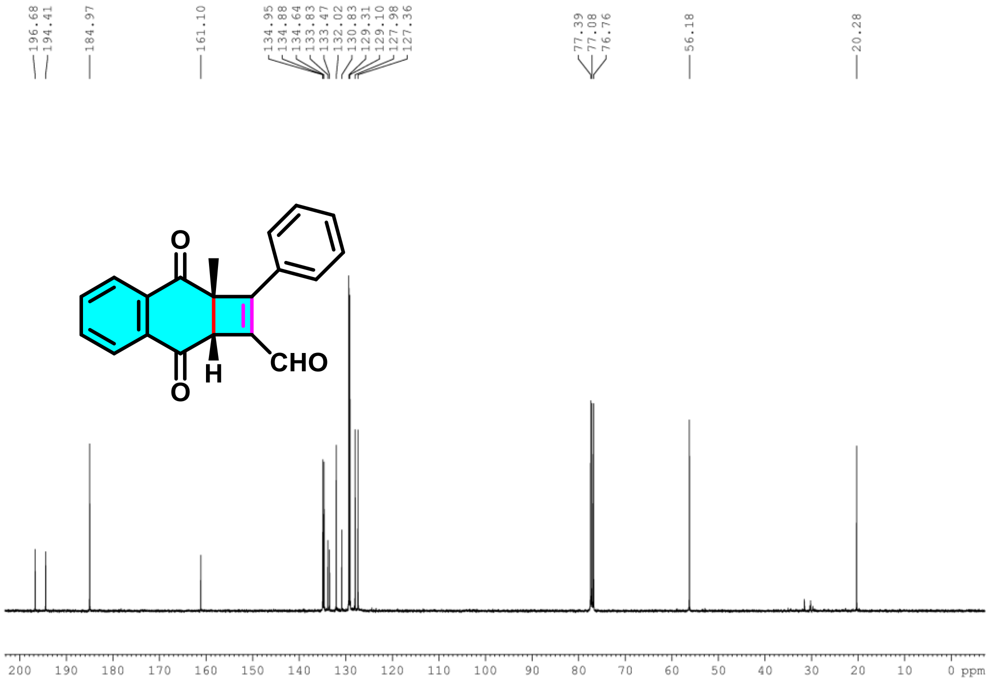

SI-Fig. 26. ^{13}C NMR spectrum of compound **3am**.

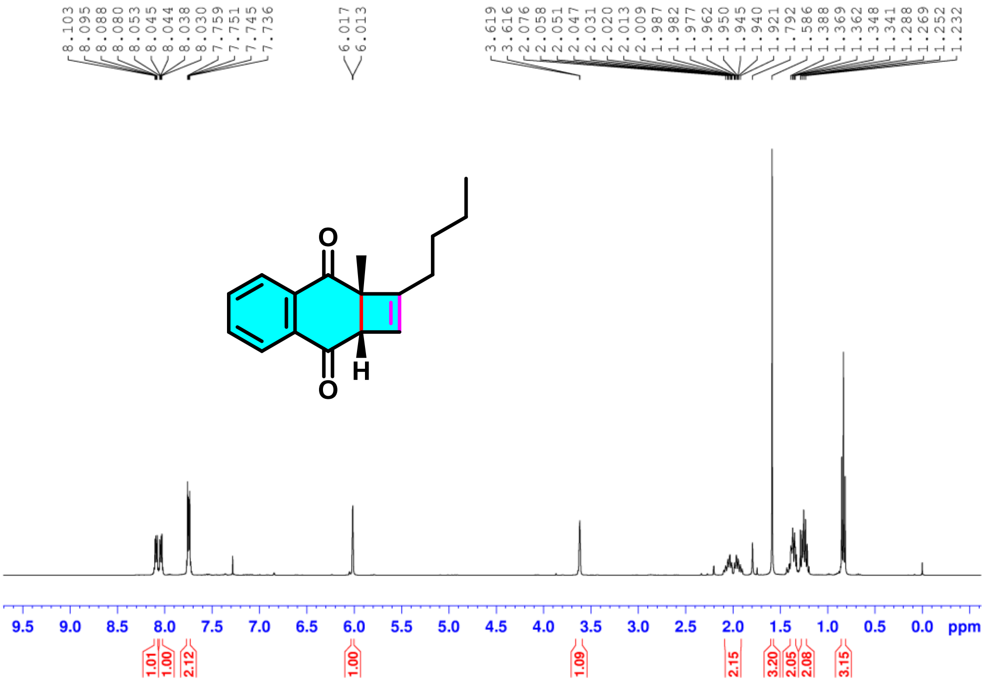

SI-Fig. 27. ^{1}H NMR spectrum of compound **3an**.

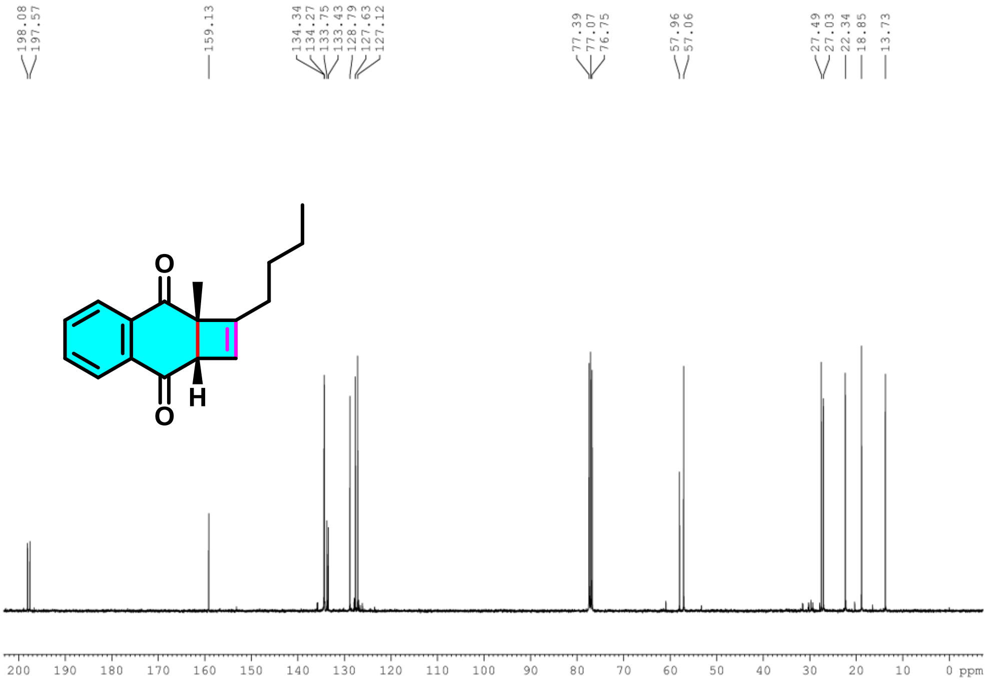

SI-Fig. 28. ^{13}C NMR spectrum of compound **3an**.

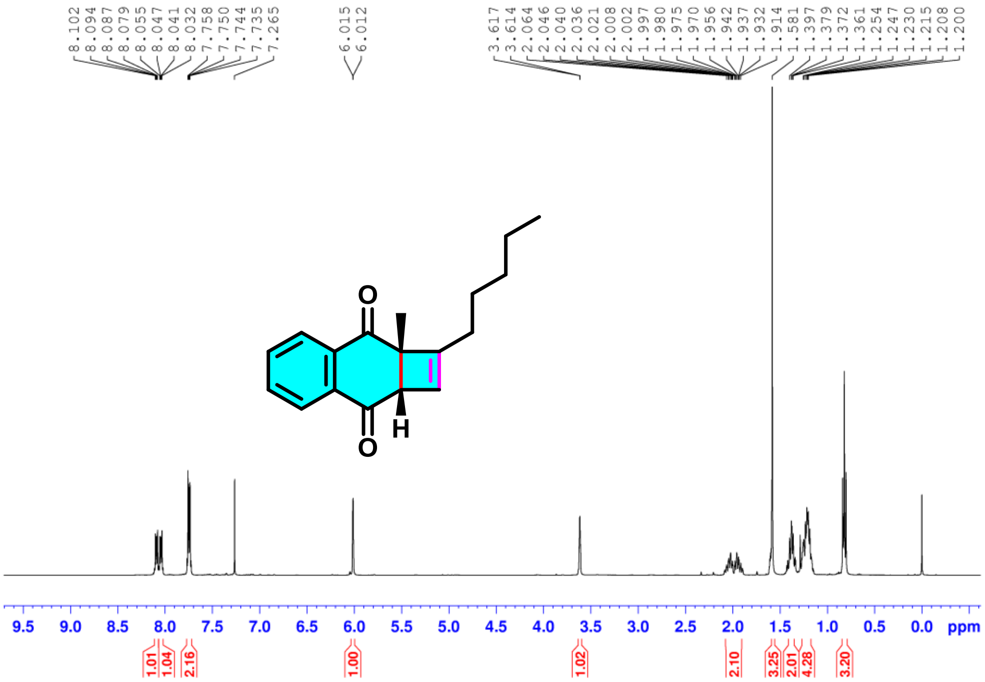

SI-Fig. 29. ^{1}H NMR spectrum of compound **3ao**.

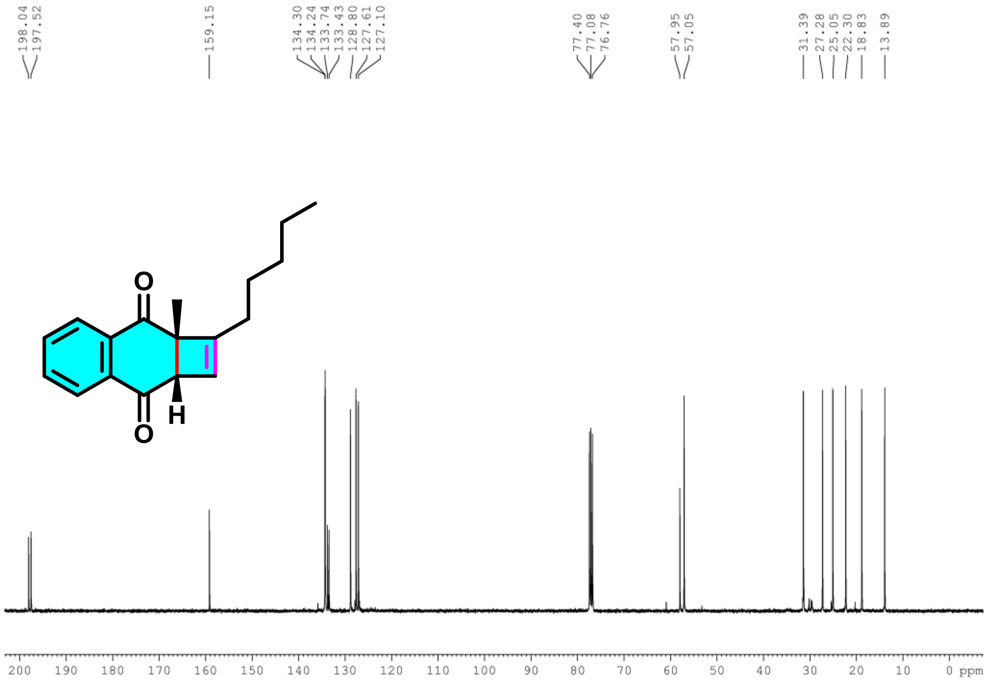

SI-Fig. 30. ^{13}C NMR spectrum of compound **3ao**.

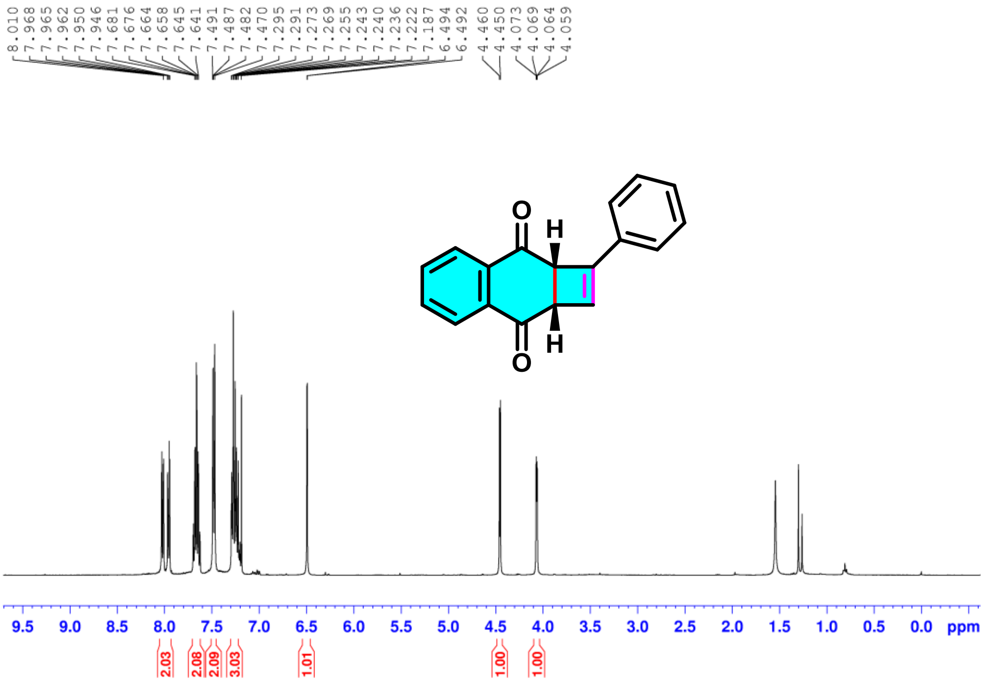

SI-Fig. 31. ^{1}H NMR spectrum of compound **3ba**.

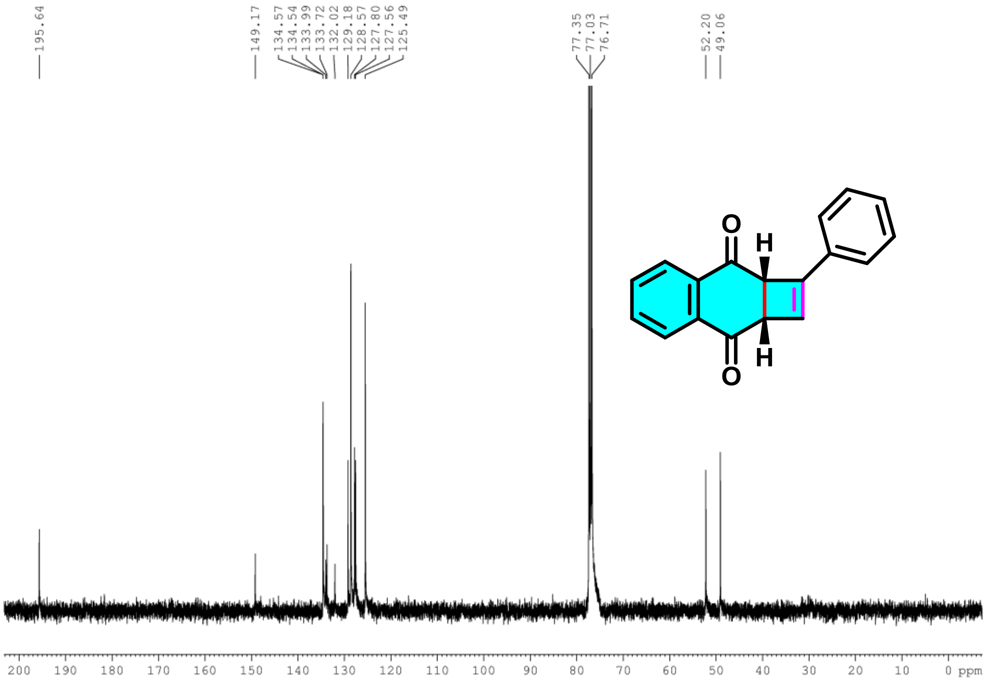

SI-Fig. 32. ^{13}C NMR spectrum of compound **3ba**.

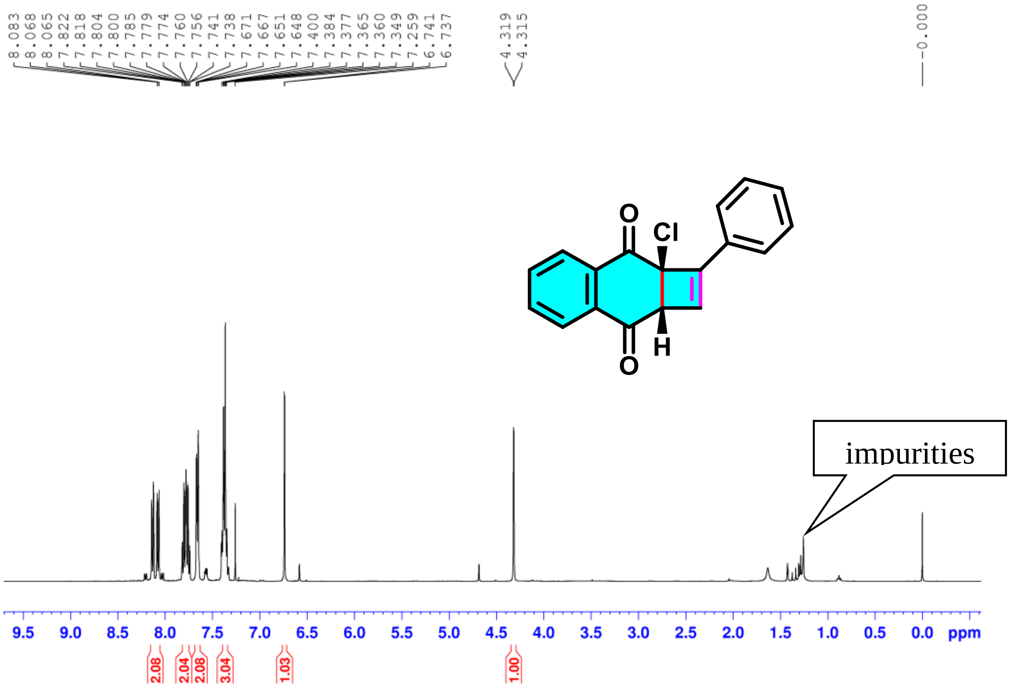

SI-Fig. 33. ^{1}H NMR spectrum of compound **3ca**.

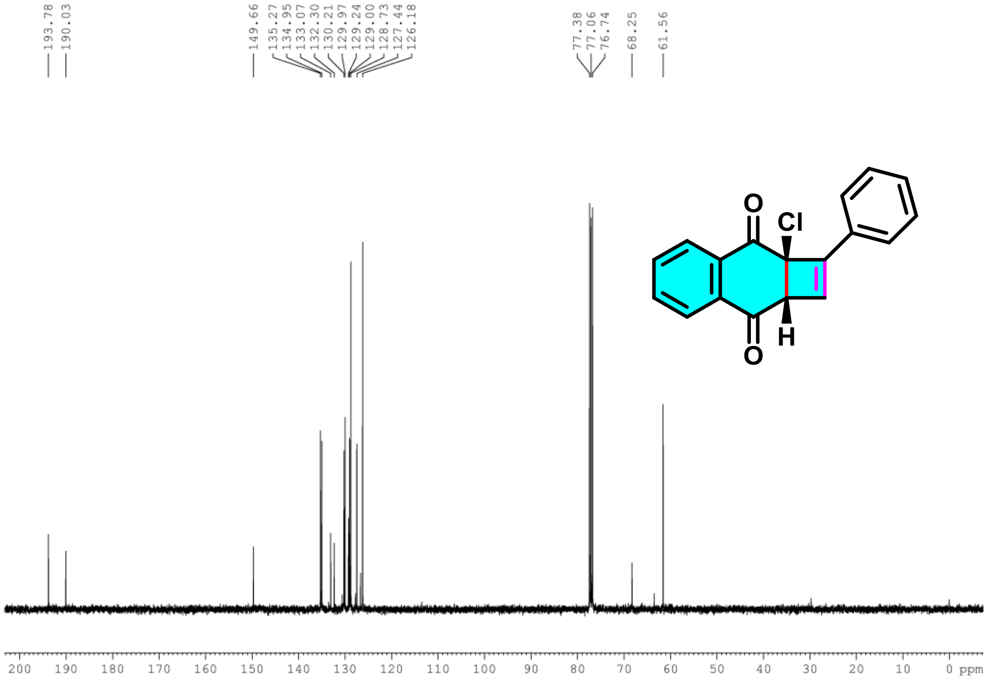

SI-Fig. 34. ^{13}C NMR spectrum of compound **3ca**.

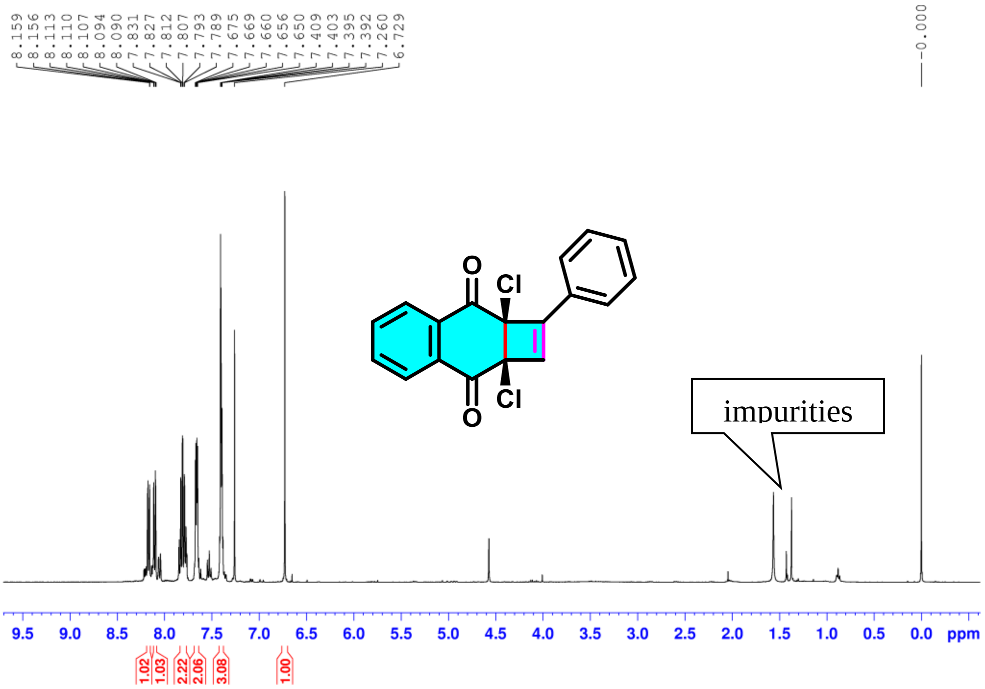

SI-Fig. 35. ^{1}H NMR spectrum of compound **3da**.

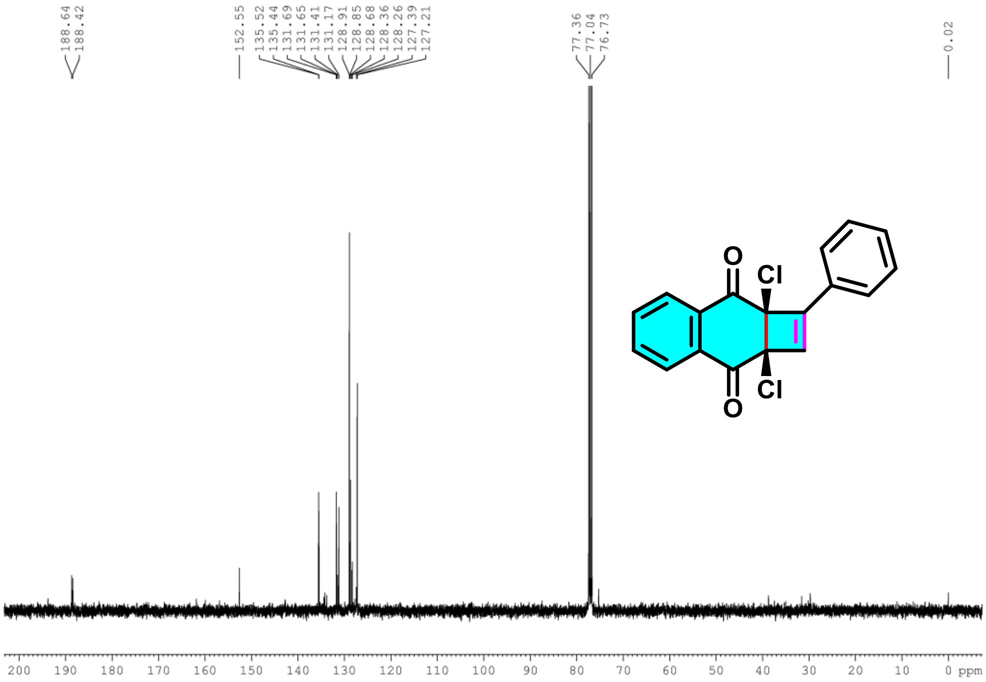

SI-Fig. 36. ^{13}C NMR spectrum of compound **3da**.

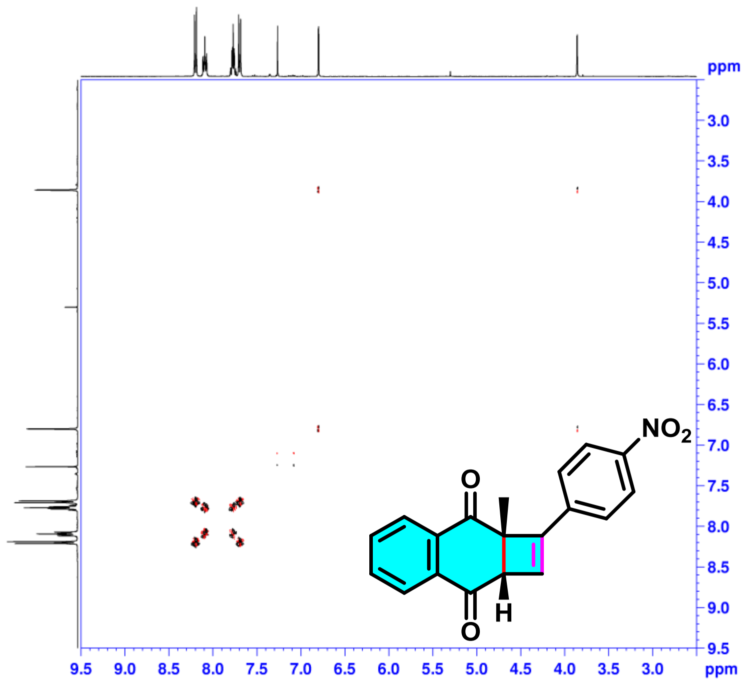

SI-Fig. 37. H-H COSY spectrum of compound **3ah**.

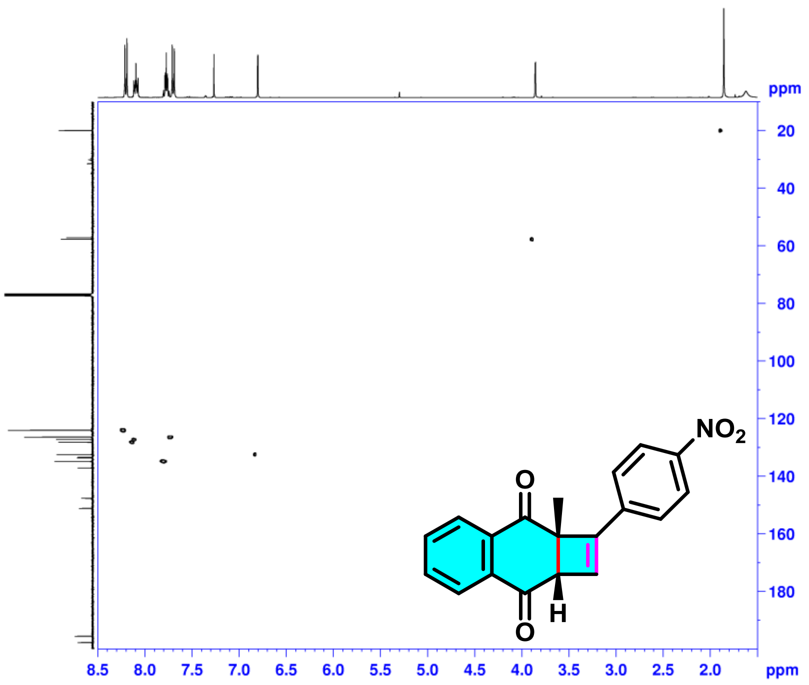

SI-Fig. 38. HSQC spectrum of compound **3ah**.

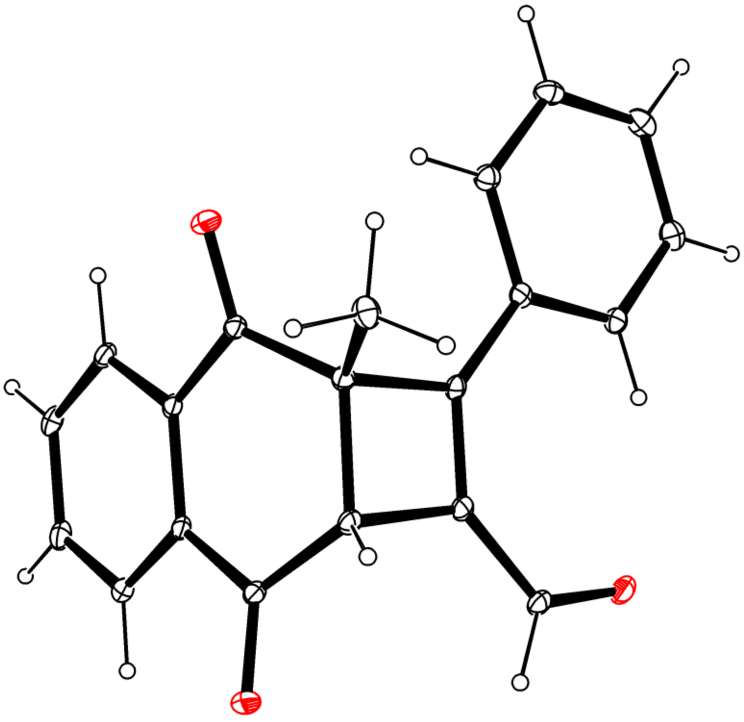

SI-Fig. 39. ORTEP diagram of crystal structures of **3am**. (CCDC-2288747)

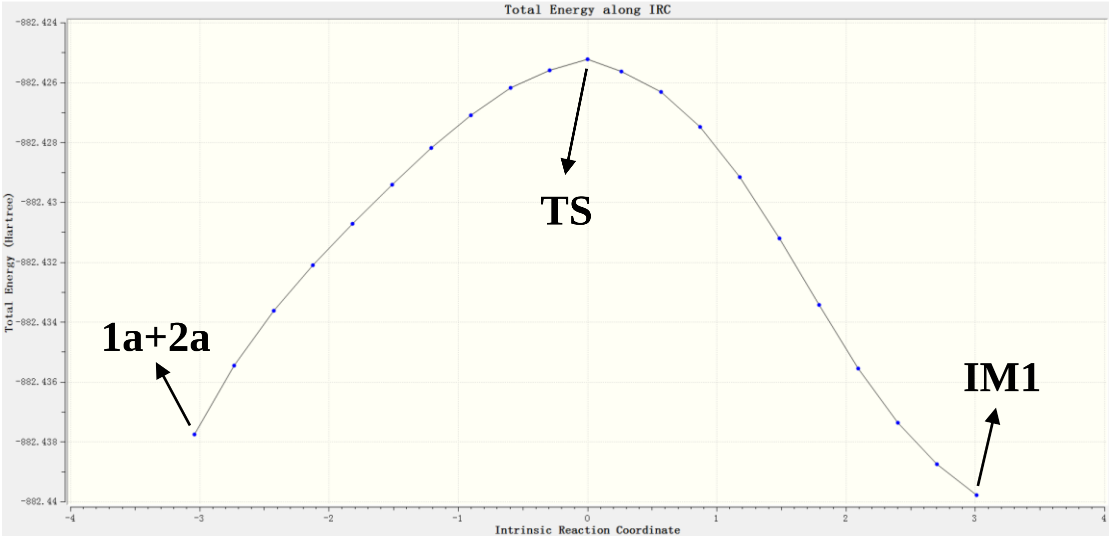

SI-Fig. 40. Total energy along IRC.

3. Cartesian coordination of stationary points

Cartesian coordination of 1a

O	5.25365600	7.37913900	-0.31739300
O	3.66061000	11.14452100	3.11906300
C	3.67093100	8.83636300	2.58971100
C	5.19660000	9.65087400	0.28243500
H	5.77476300	9.87418000	-0.61028900
C	4.80917300	10.61599800	1.13006800
C	4.01217400	10.26878900	2.35182500
C	4.87838700	8.21642300	0.48122900
C	4.08616300	7.84712100	1.69128000
C	2.93166900	8.48795400	3.71859200
H	2.62230900	9.27427200	4.39896800
C	3.76230700	6.51232000	1.92195200
H	4.09789200	5.76785700	1.20769200
C	2.61006600	7.15451300	3.94616700
H	2.03478700	6.87973500	4.82416200
C	5.11921600	12.06833700	0.94917300
H	5.67575600	12.44460300	1.81191500
H	4.19314900	12.64784200	0.90285500
H	5.69913400	12.23625000	0.04099400
C	3.02458900	6.16823900	3.04937900
H	2.77023800	5.12948800	3.23292000

****

Cartesian coordination of 1a*

O	5.25570200	7.35409700	-0.32545600
O	3.64808500	11.15581800	3.14285800

C	3.68384000	8.85172700	2.57208100
C	5.18856300	9.63445600	0.29251400
H	5.76504900	9.86857700	-0.59506100
C	4.79161900	10.60268800	1.15572200
C	4.01080000	10.28218300	2.35730000
C	4.87354100	8.24074600	0.49519600
C	4.09462800	7.85646000	1.67966800
C	2.94117600	8.49260700	3.70413600
H	2.63873900	9.28977300	4.37585000
C	3.76579400	6.51009000	1.91566000
H	4.09323700	5.75240000	1.21173200
C	2.61692800	7.16806200	3.93773500
H	2.04159400	6.89681800	4.81649000
C	5.11259700	12.05375600	0.95624000
H	5.67283100	12.43143300	1.81649900
H	4.18721200	12.63495300	0.90743400
H	5.69182600	12.21604100	0.04615800
C	3.03205900	6.17549800	3.03837800
H	2.77775000	5.13663300	3.22156500

****

Cartesian coordination of $1a^{3,*}$

O	5.26032200	7.36701600	-0.33136200
O	3.64899800	11.14991400	3.13979600
C	3.68310400	8.84781600	2.57242900
C	5.18961300	9.63743000	0.29136100
H	5.76623400	9.87417300	-0.59603300
C	4.79342100	10.60789400	1.15402800
C	4.01143700	10.27888600	2.35552800

C	4.86751400	8.25454700	0.50787400
C	4.09541900	7.84955400	1.67687600
C	2.94114700	8.48924400	3.70338600
H	2.63894000	9.28661800	4.37466100
C	3.76423800	6.50401200	1.91707300
H	4.08613500	5.73738300	1.22049200
C	2.61537800	7.16512900	3.93962000
H	2.04006500	6.89386400	4.81825400
C	5.11487700	12.05709300	0.95349300
H	5.67520400	12.43812700	1.81220400
H	4.19149600	12.64135800	0.90334200
H	5.69463000	12.21796200	0.04316100
C	3.03019100	6.17264600	3.04100400
H	2.77520800	5.13415200	3.22551200

****

Cartesian coordination of 2a

C	3.03242800	7.19982300	5.61505000
C	3.97103600	6.21327100	5.16010300
C	3.25613300	7.88836800	6.81459900
H	4.14615200	7.66631000	7.39339900
C	1.88468300	7.48147800	4.86224100
H	1.71667200	6.94546800	3.93440600
C	0.97795000	8.43660000	5.30509900
H	0.09125800	8.64981800	4.71715000
C	2.34463300	8.84218100	7.25075600
H	2.52411200	9.37163900	8.18071700
C	1.20505700	9.11837900	6.49853800
H	0.49524000	9.86389100	6.84181800

C	4.75861400	5.38386000	4.77631100
H	5.45481500	4.65073100	4.43770100

****

Cartesian coordination of TS

O	6.02460200	4.15535100	5.42363000
O	6.26654300	6.84300400	10.06554700
C	3.04647200	8.15256900	7.73235100
C	5.21224500	5.10857100	8.83619300
C	6.65614300	6.10557500	6.55838900
H	7.21226400	6.42313900	5.68127500
C	5.44740600	7.68769600	6.90614500
C	4.25600700	7.48738400	7.40659800
C	6.66152700	6.92597400	7.72630900
C	6.05234500	6.32856400	8.98905100
C	5.96696400	4.82641900	6.44728600
C	5.19696400	4.37925200	7.63838000
C	4.47749000	4.66137100	9.93798200
H	4.52281200	5.23614700	10.85679500
C	3.01425600	9.51760200	8.09604100
H	3.94223800	10.08058000	8.09618200
C	1.84000300	7.42017600	7.74460400
H	1.87937700	6.36298400	7.50201400
C	4.43064800	3.21484700	7.54762300
H	4.43948400	2.67285100	6.60788100
C	3.71353900	3.50891900	9.83513000
H	3.13560400	3.16536000	10.68699000
C	0.64038100	8.04449600	8.05144800
H	-0.28577000	7.47967800	8.03223700

C	1.82005300	10.11851600	8.46215200
H	1.80671400	11.16263600	8.75734000
C	7.80057900	7.88357700	7.99457000
H	7.48394700	8.65319500	8.69895600
H	8.14227200	8.34485000	7.06563000
H	8.63325900	7.33375700	8.44067600
C	0.63018000	9.38917900	8.42366500
H	-0.30568300	9.86891500	8.69186400
C	3.68887200	2.78478200	8.63790900
H	3.09067000	1.88224700	8.56489500
H	5.86448800	8.35071200	6.15158100

****

Cartesian coordination of IM1

O	0.76117500	-1.75514100	-2.65642500
O	1.84518200	0.06519000	2.30103300
C	-1.62824600	1.84298300	-0.24884600
C	0.95295200	-1.67079400	0.96345600
C	1.54564100	0.03477000	-1.34514400
H	2.13826800	0.36471500	-2.19284800
C	0.66222700	1.16665400	-0.75425900
C	-0.62636500	0.79270500	-0.24787800
C	1.88382300	0.54153700	-0.00805600
C	1.55108900	-0.32073600	1.18857100
C	0.94801600	-1.32837100	-1.53631000
C	0.67153300	-2.15026300	-0.32262900
C	0.69895300	-2.47393000	2.07358300
H	0.92886000	-2.07994600	3.05762300
C	-1.35700400	3.22842700	-0.21679700

H	-0.32845700	3.57551900	-0.18326400
C	-2.97436700	1.42788600	-0.24426700
H	-3.16895300	0.36044600	-0.23098000
C	0.13949000	-3.42711300	-0.48880200
H	-0.06458600	-3.77179900	-1.49692400
C	0.16187400	-3.74491900	1.90321800
H	-0.03865900	-4.36741300	2.76917700
C	-4.00958100	2.35281800	-0.27171600
H	-5.04156700	2.01908800	-0.29695200
C	-2.39091800	4.15175900	-0.17912100
H	-2.17265800	5.21340900	-0.12537400
C	3.05896200	1.46537100	0.20596500
H	2.87810600	2.10175100	1.07359700
H	3.22874300	2.08657400	-0.67620600
H	3.96260600	0.88084700	0.39885100
C	-3.71623100	3.71470500	-0.22710600
H	-4.52287800	4.44129800	-0.21621800
C	-0.11761800	-4.22200200	0.62237800
H	-0.53546800	-5.21502500	0.49262200
H	0.89745500	2.14030100	-1.19555100

****

Cartesian coordination of 3aa

O	2.35377200	-2.23870100	-2.21515900
O	0.59008000	0.65644100	1.93958700
C	0.10527200	2.80688000	-1.05852700
C	0.20076600	-1.15641800	0.48445200
C	2.24493800	-0.03629300	-1.33794200
H	3.32427200	0.02149900	-1.51202000

C	1.40604600	0.87997000	-2.20759200
C	0.96199200	1.62926800	-1.18734200
C	1.75007600	0.83694000	-0.13341400
C	0.83591000	0.14291300	0.86505200
C	1.81731200	-1.48306500	-1.43182900
C	0.66339700	-1.92555700	-0.59181800
C	-0.85921300	-1.62435700	1.26460600
H	-1.18769300	-1.01794500	2.10172900
C	-0.37857700	3.45098300	-2.20568800
H	-0.11009000	3.06763500	-3.18601400
C	-0.23644100	3.30887700	0.20258900
H	0.12085800	2.80667600	1.09640700
C	0.06482600	-3.15359800	-0.87797800
H	0.45659000	-3.73348600	-1.70689500
C	-1.46152100	-2.83853200	0.96428000
H	-2.28984700	-3.19532100	1.56763500
C	-1.04791300	4.43496300	0.30942400
H	-1.30923300	4.81622500	1.29115200
C	-1.18830400	4.57193000	-2.09411100
H	-1.55716100	5.06347200	-2.98850900
C	2.85239100	1.61596300	0.57452600
H	3.49702000	0.93887600	1.14241400
H	2.42592800	2.34000200	1.27038900
H	3.46026500	2.14755100	-0.16213300
C	-1.52446300	5.06809000	-0.83410000
H	-2.15613900	5.94620100	-0.74738200
C	-0.99857900	-3.60487300	-0.10797000
H	-1.46742600	-4.55654700	-0.33603200
H	1.25518900	0.88883700	-3.28158700

****
